# Inclusion of a Phytomedicinal Flavonoid in Biocompatible Surface-Modified Chylomicron Mimic Nanovesicles with Improved Oral Bioavailability and Virucidal Activity: Molecular Modeling and Pharmacodynamic Studies

**DOI:** 10.3390/pharmaceutics14050905

**Published:** 2022-04-21

**Authors:** Mohamed Y. Zakaria, Paris E. Georghiou, Joseph H. Banoub, Botros Y. Beshay

**Affiliations:** 1 Department of Pharmaceutics and Industrial Pharmacy, Faculty of Pharmacy, Port Said University, Port Said 42526, Egypt; dr_m_yehia@live.com; 2 Department of Chemistry, Memorial University of Newfoundland, St. John’s, NL A1B 3X7, Canada; joe.banoub@dfo-mpo.gc.ca; 3 Fisheries and Oceans, Canada Science Branch, St. John’s, NL A1A 5J7, Canada; 4 Department of Pharmaceutical Sciences (Pharmaceutical Chemistry), College of Pharmacy, Arab Academy for Science, Technology and Maritime Transport, Alexandria 21913, Egypt; botros_beshay@aast.edu

**Keywords:** morin hydrate, oral chylomicron, flavonoid, statistical optimization, docking, ADME, virucidal activity, MERS-CoV, pharmacokinetic study

## Abstract

Morin hydrate (MH) is a widely-used Asian phytomedicinal flavonoid with a wide range of reported therapeutic activities. However, MH has limited oral bioavailability due to its low aqueous solubility and intestinal permeability, which in turn hinders its potential antiviral activity. The study reported herein was designed to encapsulate MH in polyethyleneglycolated (PEGylated) chylomicrons (PCMs) and to boost its antiviral activity and biological availability for oral administration using a rat experimental model. The PEGylated edge activator combined with the conventional components of chylomicrons (CMs) amplify the transport of the drug across the intestine and its circulation period, hence its therapeutic impact. The implementation of variables in the in vitro characterization of the vesicles was investigated. Using Design Expert^®^ software, a 2^4^ factorial design was conducted, and the resulting PCM formulations were fabricated utilizing a thin-film hydration technique. The efficacy of the formulations was assessed according to their zeta potential (ZP), entrapment efficiency percentage (EE%), amount of drug released after 8 h (Q8h), and particle size (PS) data. Formulation F9, which was deemed to be the optimal formula, used compritol as the lipidic core together in defined amounts with phosphatidylcholine (PC) and Brij52. Computer-aided studies revealed that MH alone in a suspension had both diminished intestinal permeability and absorption, but was enhanced when loaded in PCMs. This was affirmed by the superiority of formulation F9 results in ex vivo permeation and pharmacokinetic studies. Furthermore, formulation F9 had a superior safety profile and antiviral activity over a pure MH suspension. Molecular-docking studies revealed the capability of MH to inhibit MERS-CoV 3CL^pro^, the enzyme shown to exhibit a crucial role in viral replication. Additionally, F9 suppressed both MERS-CoV-induced histopathological alteration in lung tissue and resulting oxidative and inflammatory biomarkers. Collectively, the results reported herein affirmed the potential of PCMs as nanocarriers for the effective oral administration of MH as an antiviral.

## 1. Introduction

Middle East Respiratory Syndrome (MERS), or “Camel flu”, is one of the most virulent infections that strikes the pulmonary tract [1]. It was first identified in Saudi Arabia in 2012 [2]. The coronavirus MERS-CoV is responsible for the infection. MERS induces grave respiratory distress in humans, is highly contagious and is spread between humans in close contact, and has a high mortality rate [1,2]. Nevertheless, its risk to the global population in recent years has been considered to be relatively low. The evolution of several infectious coronavirus outbreaks and pandemics, including MERS-CoV and SARS-CoV-2 (COVID-19), have resulted in the screening [3] and testing [4] of many approved and developmental drugs for controlling MERS-CoV infections. It is known that the replication cycle of MERS-CoV can be disrupted by suppressing the production of the nonstructural papain-like protease, PL^pro^ [5]. PL^pro^ is a protease characterized by its vital and versatile range of tasks that are incorporated in the coronavirus viral replication cycle. It not only affects viral cellar entry, but also is essential for the fragmentation of the pp1ab and pp1a polyproteins, forming mature nonfunctional proteins [5]. Thus, investigation of drugs that can exploit the antiviral activity via the inhibition of PL^pro^ is of significant interest.

Several attempts at employing natural-product-derived drugs as potential leads for reducing MERS-CoV infection and its consequences have been reported. As a result of their chemical and structural diversity, as well as the comparable biosafety that many natural compounds can offer, they have long been considered to be sources of various medical remedies, especially against viral infections and their consequences [6]. Resveratrol, a widely used natural phytochemical found in many plants, has been reported to elicit a potent anti-MERS-CoV activity, and to be capable of resolving its inflammatory and oxidative distress. It is classified as a Class II drug in the Biopharmaceutics Classification System (BCS) [7]. For the study reported herein, we employed a structurally related natural product to resveratrol; namely, morin hydrate (MH), a flavonoid (2′,3,4′,5,7-pentahydroxyflavone) derived from the white mulberry tree, *Morus alba* L. [8]. MH has shown anticancer, antiapoptotic, antioxidant, and anti-inflammatory activities [9]. In addition, MH has displayed a neuroprotective impact in Parkinsonism [10]. Despite the observed inhibitory effect of MH on the autophagy pathway [10] and the NF-κB signaling pathway [11], a comprehensive molecular mechanism of its action has not been investigated. The antiviral activity of MH against an influenza virus was also recently reported [12]. However, MH is categorized only as a Class IV BCS drug. This is due to its poor permeability and poor solubility, predisposing it to hindered bioavailability [10]. A comprehensive review of MH’s versatile biological and pharmacological potential was published in 2021 [13].

The evolution of natural-lipid-based nanovesicles that possess structural resemblances to biological chylomicrons (CMs) are now well recognized as potential candidates for organ-targeted drug delivery [14], along with other liposomes [15]. They have the advantages of being biologically compatible drug nanocarriers. In synthetic lipoproteins, lipids are incorporated such that they conform to the structure of a vesicular shell and core [16]. Moreover, they can be regarded as being hybrids of nanoemulsions and nanovesicles. Despite being capable of shielding drugs, the diminished stability, restrained encapsulation efficiency, and scale-up problems found in conventional vesicular systems such as liposomes and niosomes have prompted the evolution of de novo vesicular systems [17]. Some of these restrictions can be mitigated by the use of CMs, which offer biocompatible platforms for the oral delivery of lipophilic drugs. CMs, which are involved in the transport of dietary lipids from the intestines to other locations in the body, are ultralow-density lipoproteins. They are composed of triglycerides (85–92%), phospholipids (6–12%), cholesterol (1–3%), and proteins (1–2%). CMs encompass two crucial components; namely, a lipid core that is hedged with phospholipid outer layers that confer vesicle stability, and hence can be fabricated without the need of additional surfactants or stabilizers. Lipophilic moieties can be enclosed in the lipid core of the CMs, resulting in diminished drug leakage and extended release. Furthermore, the surfaces of CMs can be PEGylated to provide boosted steric stabilization and extensive circulation duration [18]. The features described in the foregoing, therefore, could result in enhanced bioavailability of MH, and thus also lessen its dosage requirements.

In the study reported herein, the improvement in the biological activity and oral availability of MH was achieved by tailoring the compositional formulae of polyethyleneglycolated (PEGylated) chylomicrons (PCMs). This was achieved by considering several variables in a 2^4^ full-factorial design. The optimum PCM formula was investigated for its morphology, and a computer-aided absorption, distribution, metabolism, elimination, and toxicity (ADMET) study was conducted to predict the characteristics of the drug, which was confirmed by ex vivo and pharmacokinetic studies. Furthermore, a molecular modeling study was conducted to provide potential insights into the proposed anti-MERS-CoV site of action of MH. Finally, the boosted antiviral and anti-inflammatory activities of MH were confirmed by comparing the efficacy of the optimal MH-loaded PCM system with that of a suspension of MH in a MERS-CoV-challenged animal model.

## 2. Materials and Methods

### 2.1. Materials

The morin hydrate (MH), egg yolk L-α-phosphatidylcholine (PC) (lecithin), and triolein were purchased from Sigma-Aldrich, Inc., St. Louis, MO, USA. The Brij52 (polyoxyethylene (2) cetyl ether) was obtained from BASF Co. (Florham Park, NJ, USA). The compritol 888 (glyceryl behenate) was acquired as gift from Gattefosse (Saint-Priest, France). The sodium chloride, potassium dihydrogen orthophosphate, methanol, sodium hydroxide, magnesium chloride, chloroform, and absolute ethanol were purchased from El-Gomhouria Chemical Co., Cairo, Egypt. The dialysis membranes (Spectra/Pore^®^, cut-off 12,000–14,000) were purchased from Spectrum Laboratories Inc., Rancho Dominguez, CA, USA). All chemicals and solvents were of analytical grade and were used as received. The specific ELISA kits were obtained from eBioscience Co, San Diego, CA, USA. The Vero cells (African green monkey kidney cells, Vero C1008 (Vero 76, clone E6, Vero E6)) were purchased from Viromed Laboratories (Minnetonka, MN, USA).

### 2.2. Tailoring of Morin Hydrate (MH)-Charged Polyethyleneglycolated Chylomicrons (PCMs) and Preparation of the MH Dispersions

MH-charged PCMs were prepared using the thin-film hydration technique with slight modification [19]. For the fabrication of the PCMs, MH (20.0 mg) was dissolved in a 2:1 chloroform:methanol mixed solvent (10 mL), with the lipid core (compritol, or triolein) used in either 20.0 or 60.0 mg amounts, together with 20.0 or 40.0 mg amounts of PC, Brij52 (10.0 or 30.0 mg), and cholesterol (10.0 mg) in a round-bottom flask covered with aluminum foil to protect the components from light decomposition (Table 1). The organic solvent was then completely removed under reduced pressure using a rotary evaporator for 30 min at 60 °C. The resulting dry film was then suspended in phosphate buffer solution (PBS; 10 mL) for 45 min at 60 °C, a temperature that exceeded the transition temperature of the lipid phase (Tc) [20]. In order to reduce the particle size of the resulting PCM dispersions, an ultrasonicator (Elmasonic, Model LC 60/H Germany) was employed at room temperature for a further 10 min.

The MH dispersion solutions that were used as controls were prepared by room-temperature sonication of 20.0 mg amounts of MH (mol wt. 321.05) in 10.0 mL volumes of PBS. All preparations were stored at 4 °C in amber-colored glass tubes to prevent potential ambient light degradation of the MH [21,22].

### 2.3. In Vitro Investigation and Optimization of the Morin-Hydrate-Charged PCMs

#### 2.3.1. Determinations of the Entrapment Efficiency Percentage (EE%)

The entrapment efficiency percentages (EE%) of MH in the fabricated PCM dispersion were determined in triplicate as follows: 1.00 mL aliquots of the MH-charged PCM (comprising 2.0 mg of the drug) were first dispersed into distilled water (5.0 mL), agitated for 2 min, and then centrifuged (Beckman Instruments, Fullerton, CA, USA) at 15,000 rpm and with cooling at 4 °C for 1 h [17]. The deposited vesicles were rinsed twice with distilled water and then recentrifuged for 30 min. The vesicles in methanol were then sonicated at room temperature for 2 h, and the embedded MH concentrations within the vesicles were analyzed by UV spectrophotometry at λ_max_ 375 nm versus methanol as the blanks. The EE% values were calculated using the formula:

**EE% of MH entrapped = (amount of MH enclosed/overall amount of MH) × 100**

#### 2.3.2. Determinations of Zeta Potential (ZP), Particle Size (PS), and Polydispersity Index (PDI)

The particle (or vesicle) size (PS), zeta potential (ZP), pH, and polydispersity index (*PDI*) of the MH-charged PCM preparations (Table 2) were determined in triplicate [19] using a Zetasizer ZS (Malvern Instruments, Malvern, UK). The MH-charged PCMs (0.10 mL) were admixed with distilled water (10.0 mL) in a glass culture tube and vortexed for 5 min. The particle sizes and their scattering surrounding the mean (including those of the pure-MH dispersion, shown in Supplementary Materials Figure S1) were determined at 25 °C using dynamic laser scattering with a 45 mm focus lens and a beam length of 2.4 mm. The same instrument was utilized for the ZP measurements of the electrophoretic mobility of the particles in the electric field.

#### 2.3.3. In Vitro Morin Hydrate Release Experiment

In a 2.5 cm diameter 10 cm glass cylinder fitted at the bottom with a presoaked cellulose membrane, we added 1.00 mL of the MH-charged PCM dispersions (comprising 1.0 mg of MH) produced from the dilution of 1.00 mL of the stock preparations to 1.00 mL of Sorensen phosphate buffer (pH 7.4). The glass cylinder was carefully fixed onto the shaft of the dissolution tester (Copley, DIS 8000, Nottingham, UK) and was mounted in 900 mL of the same Sorensen phosphate buffer at 37 ± 0.5 °C and a speed of 50 rpm [23]. Equal-volume aliquot samples were removed at scheduled time intervals. To maintain a constant volume and a constant sink condition, equal volumes of freshly prepared dissolution medium were utilized to replace the withdrawn samples. The percentages of MH released were assessed spectrophotometrically at 372 nm and were measured in triplicate.

### 2.4. Experimental Design and Choice of the Optimal Morin-Hydrate-Charged PCMs

A 2^4^ factorial analysis [24] was undertaken using Design Expert^®^ Version 7 (Stat Ease, Inc., Minneapolis, MN, USA) to explore the consequences of the variation of several formulation aspects of the PCMs and for the statistical analysis of the outcomes. Sixteen runs were acquired from the constructed design. Four factors that were the independent variables were considered: lipid core type (A), lipid core amount (B), PC amount (C), and Brij52 amount (D). The four values; namely, EE% (Y1), PS (Y2), ZP (Y3), and Q8h, (Y4) were selected as the dependent variables. The choice of the optimal MH-charged PCM formulation was established according to the greatest Q8h EE%, ZP, and minimal globule size values. ANOVA statistical analyses were used to consider the main effects and the significance of each of the variables under examination. The optimal formulation with the superior desirability value was selected for further investigations [25].

### 2.5. In Vitro Exploration of the Optimum Morin-Hydrate-Charged Polyethyleneglycolated Chylomicron (PCM) Formula

#### 2.5.1. Lyophilization of the Optimized PCM Formulation

Lyophilization was conducted using a freeze-dryer (Alpha 2–4, CHRIST Osterodeam Harz, Germany) for the solidification of the optimized MH-charged PCM formula. Mannitol (5% *w*/*v*) was used as the cryoprotectant to impede the lysis of the vesicles. The PEML suspension was stored overnight at −80 °C and was dried for 24 h under vacuum [18]. For further assessments, the prepared emulsomal powder was kept in a desiccator in tightly closed amber-colored glass tubes.

#### 2.5.2. Differential Scanning Calorimetry (DSC)

The thermal behavior of each of pure MH, cholesterol, plain optimum formula, and MH-loaded PCMs were determined by DSC (DSC-50, Shimadzu, Kyoto, Japan). Purified indium (99.9%) was used to calibrate the equipment. The temperature was raised at 10 °C/min in a temperature range of 20–400 °C under nitrogen [23].

#### 2.5.3. Transmission Electron Microscopy (TEM)

Confirmation of the optimum PCM formulation was evaluated by TEM using a JOEL JEM 1230 instrument. A carbon grid with a copper coating was adhered to the stained vesicles’ dispersion and was left to dry to obtain a thin film. The resulting copper sheet was introduced into the TEM [26].

#### 2.5.4. Impact of Storage on the In Vitro Properties of the Optimized F9 Formula

This experiment was conducted by preserving samples of the optimized formulation at 4 °C and at 25 °C for a period of 3 months. At 0 and at 3 months, the samples from the formulation were withdrawn and the storage effect was examined with respect to the PS, EE%, Q8h, and ZP values, and via one-way ANOVA analysis, the significance of the results of the parameters under examination at a level of *p* < 0.05 was investigated [27].

### 2.6. Ex Vivo Drug Gut-Permeation Study

A comparative ex vivo intestinal transport study between the drug suspension and the optimized formula was conducted using the noneverted gut sac technique [10]. Diethyl ether was utilized to anesthetize 12 overnight-fasted male Wistar rats with weights between 180 and 260 g; they were then terminated via cervical dislocation. The small intestine from each animal was then excised and sliced into sections 5 cm in length. The sections were then rinsed using normal saline. Krebs–Ringer solution (~2 mL) was utilized to irrigate the intestinal segments, followed by sac formation by closing the lower portion of the segments using a thread. Each sac was filled with a sample of the pure MH suspension (estimated to comprise 5.0 mg of MH) or with the optimized F9 formulation (estimated to comprise 5.0 mg of MH). Finally, each sac was allocated in an organ path packed with 50 mL of Krebs–Ringer solution after the lower portion of each sac was tightly sealed with a thread. The organ path was preserved under continuous stirring at 100 rpm at 37 ± 0.5 °C, and with continuous aeration with an 95:5% oxygen: CO_2_ mixture. To estimate the permeated amount of MH, 2.00 mL samples were withdrawn at prescheduled time intervals (15, 30, 45, 60, 75, and 90 min). To maintain a constant total volume, the organ path was compensated by the addition of equal volumes of fresh Krebs–Ringer solution after each sample for analysis had been withdrawn. The amounts of MH that had permeated through the intestinal segment were determined via HPLC using a Hitachi LaChrom Elite instrument equipped with a Model Series L-2000 organizer box, L-2300 column oven, L-2130 pump with built in degasser, Rheodyne 7725i injector with a 20.0 mL loop, and a L-2455 photodiode array detector (PAD). The mobile phase used was aqueous 10 mM potassium dihydrogen phosphate (pH 5.0) and acetonitrile (60:40, v/v) with a flow rate of 1.0 mL/min. A wavelength of 375 nm was used for the determinations [28].

The apparent permeability (**Papp**) of PCM versus MH suspension (in units of cm min^−1^) was estimated using the following formula:

**Papp = (F/A) × (C_0_)**

where **F** is the permeation flux, **C_0_** is the initial concentration, and **A** is the total surface area of the ileum.

### 2.7. In Vitro Virucidal Activity Assessments

#### 2.7.1. Dimethylthiazol-2-yl-2,5-diphenyltetrazolium Bromide (MTT) Cytotoxicity Assay (TC_50_)

Estimation of the cytotoxic activity of the formulation samples with Vero E6 cells was conducted using dimethylthiazol-2-yl-2,5-diphenyltetrazolium bromide (MTT) [29] with minute modifications. Dulbecco’s Modified Eagle’s Medium (DMEM) was utilized for sample dilutions. The stock solutions of the investigated formulations were employed as solutions in 10% DMSO in double-distilled H_2_O. Cells were harvested in 96-well plates (100-µL/well at a density of 3 × 10^5^ cells/mL) and were incubated for 24 h at 37 °C in 5% CO_2_. Afterwards, various amounts of the investigated compounds were added to the cells, and each experiment was performed in triplicate. After an extra 24 h, cell monolayers were dislodged from the supernatant and then rinsed 3 times with sterile phosphate buffer saline (PBS). MTT solution (20.0 µL of 5.00 mg/mL stock solution) was added into each well, and the cells were incubated for 4 h at 37 °C, followed by aspiration of the medium. The formed MTT-derived formazan product crystals were dissolved in each plate by the addition of 200 µL of acidified isopropanol (0.040 M HCl in absolute isopropanol = 0.073 mL HCL in 50.0 mL isopropanol). The absorbances of the formazan solutions were assessed at λ_max_ 540 vs. the 620 nm reference wavelength, using a multiwell plate reader. The cytotoxicity percentages related to the untreated cells were computed via GraphPad Prism software (version 7.01) using the following formula:

**%Cytotoxicity = (absorbance of cells without treatment − absorbance of cells with treatment) × 100 (absorbance of cells without treatment)**

The concentration that showed 50% cytotoxicity (TC50) was assessed via the plot of %cytotoxicity vs. sample concentration [30,31].

#### 2.7.2. Plaque Assay

An assay of plaque lodged on Vero E6 cells was employed in the investigation of the MER-CoV viral titers. As employed in previous studies, Vero E6 cells (105 cells/mL) were refined for 24 h at 37 °C in six-well plates [32]. The dilution of MERS-related coronavirus isolate NRCE-HKU270 (Accession Number: KJ477103.2) yielded 103 PFU/well. Together with the predetermined safe concentration of the investigated samples, incubation was conducted for 1 h at 37 °C. After the growth medium was aspired, the cells were challenged with virus (100 µL/well) with the investigated formulation samples for 1 h as a suitable contact time for permitting the viral adsorption. Afterward, cell monolayers were subjected to 3.0 mL of DMEM after first being combined with 2% agarose and the investigated samples. The plates were set aside for solidification, and then were incubated at 37 °C until the emergence of viral plaques (3 to 4 d). The cells were supplemented with 10% formalin for 2 h and then with 0.1% crystal violet in distilled water for staining. For comparison, the control wells, denoted as untreated virus, were incubated with Vero E6 cells, and the percentage of reduction in plaque constitution related to control wells (i.e., **%Inhibition**) was computed using the following formula:

**%Inhibition = [viral count (untreated) − viral count (treated)]/[viral count (untreated)] × 100**

### 2.8. Molecular Modeling Computational Analysis

#### 2.8.1. Ligand Preparation, Protein Preparation and Docking Process

The structure of the MH was retrieved in .sdf format from the PubChem database, and was energetically minimized using the “Generate Conformations” tool in Discovery Studio (DS) 5.0 client (Dassault Systèmes BIOVIA. Discovery Studio Modeling Environment, Release 2017; Dassault Systèmes, San Diego, CA, USA). The X-ray coordinates of the MERS-CoV 3CL^pro^ with the co-crystallized experimental ligand GC813 (resolution 1.55A) **PDB 5WKK** [33] was downloaded from the Protein Data Bank (https://www.rcsb.org, accessed on 20 December 2021). The addition of missing atoms/chains and the removal of water molecules in the protein structure was employed via the “Prepare Protein” protocol in DS. The “Prepare Protein” algorithm was used for the protonation of amino acid residues at the target pH 7.0 ± 2.0. Defining of the conjugation site was conducted by selecting the conjugation sphere and wrapping the co-crystalized ligand. CDOCKER [34] was utilized as the grid-based docking program to fit the active compounds in the 3CL^pro^ binding site using the default parameters. The dominant fitted pose of the docked MH was characterized on the basis of the CDOCKER energy (-CDE).

#### 2.8.2. Investigation of the In Silico Predictive Absorption, Distribution, Metabolism, Elimination, and Toxicity (ADMET) for Morin Hydrate

Discovery Studio (DS) 5.0 client was employed to determine the computer-aided ADMET parameters. The obtained results were based on the chemical structure of the MH and involved the determinations of pharmacokinetics parameters. These included aqueous solubility levels, absorption levels, atom-based Log P98, 2D polar surface area, blood–brain barrier levels, and hepatotoxicity probability as determined by cytochrome P450 2D6 (CYP PROB) levels.

### 2.9. In Vivo Characterization of the Selected Morin Hydrate (MH)-Loaded PCM

#### 2.9.1. Experimental Animals and MERS-CoV Viral Infection

A total of 40 10-week-old female C57BL/6 mice weighing 150 ± 10 g were obtained from the Biological Production Unit (BPU) of the Theodore Bilharz Research Institute (Giza, Egypt). The mice were sheltered in standard polypropylene cages for seven days while preserving the optimum laboratory circumstances regarding humidity, temperature, and light, with 12 h of dark/light cycles. The mice had a free access to diet and water *ad libitum* in order to minimize the variation [23]. The Research Ethics Committee of the Faculty of Pharmacy at Port Said University approved the experimental protocol. Viral solutions (50 µL) encompassing 10^5^ plaque-forming units (pfu) of 50% tissue culture infectious dose (TCID50) of MERS-CoV were intranasally inoculated in the experimental animals [3]. The animals were subcategorized into four groups (10 mice in each group) and were kept under continuous monitoring of their body weights and survival rates. The animals, which were randomly assigned into different groups, in turn received different dosage regimens (all doses in PBS) as follows:

***Group 1***: Control (uninfected): saline (2.0 mL/kg) intraperitoneally (i.p.) once, then administered orally saline at a 5.0 mL/kg daily dose.

***Group 2***: MERS-infected (untreated): intranasally infected with 105 pfu/50 μL of virus.

***Group 3***: MH-suspension-treated after infection with an oral dose of 10 mg/kg (12) once daily for 6 d.

***Group 4***: MH-PCM-treated after infection with an oral dose of 10mg/kg once daily for 6 d.

#### 2.9.2. Viral Titer Investigation in Acutely Infected Lungs by Adopting a Plaque Assay

Preceding viral infection, the animals were anesthetized with urethane (1.6 g/kg) at day 3 and day 6, followed by their termination (on day 6) by intraperitoneal overdose of sodium pentobarbital (30–50 mg/kg). The lungs of the animals were then dislocated and assembled immediately. They were dried with tissue paper after first being washed with normal saline. The weights of the lungs were recorded, and then the lungs were treated with physiologic saline (1.0 mL) and protease inhibitor (20 mM Tris-HCl, 150 mM NaCl, 1% Triton X-100, 0.1% SDS, Roche complete ULTRA Tablet). They were then homogenized using a glass tissue homogenizer (MPW-120 homogenizer, Bitlab, Shanghai, China), and finally centrifuged for 10 min at 10,000 rpm. The aspirates were frozen at −20 °C or at −80 °C until the investigations [35]. Serial dilutions using DMEM of the lung homogenate were employed for the infection with Vero E6 cells at 37 °C for 2 h. The acquired Vero monolayers were seeded in 12-well plates and then challenged with serially diluted homogenate containing infectious virus (200 μL/well), and assayed as previously described.

#### 2.9.3. Histopathological Investigations

The excised tissues of the lung were preserved in 4% formalin and were then embedded in paraffin. The preserved tissues were sliced into 5 µm sections, placed onto glass slides, then stained with hematoxylin and eosin (H&E) stain. The stained tissues were examined using an Olympus CX41 (Olympus, Tokyo, Japan) microscope [36]. According to the grade of the pathological changes and inflammatory consequences visualized in the investigated tissues, they were scored as: ***0*** for no changes; ***1*** for mild changes; ***2*** for moderate changes; and ***3*** for severe changes.

#### 2.9.4. Assessment of Oxidative and Inflammation Biomarker Induced by MERS-CoV Infection

The bronchial alveolar lavage fluid (BALF) was harvested after the animals were anesthetized with urethane (1.6 g/kg), and then the BALF was processed for further investigation of cytokines and oxidative biomarkers [37]. In accordance with previously conducted studies, the BALF samples were aspirated via a cannula that invaded the trachea, while the right lung was rinsed 5 times, with 1.0 mL physiological saline followed by collection through a tracheal cannula. The resulting supernatant following the centrifugation of the BALF at 2500 rpm at 4 °C for 10 min was then collected and kept at −70 °C for further investigations. For the investigation of superoxide dismutase (SOD) activity in the BALF [38], a SOD ELISA mouse kit (eBioscience Co, San Diego, CA, USA) was employed according to the instructions given by the manufacturer. In addition, estimation of interleukin-6(IL-6), interferon-gamma (IFN-γ), tumor necrosis factor (TNF)-α, and interleukin-4 (IL-4) was conducted using precise ELISA kits (eBioscience Co., San Diego, CA, USA) following the instructions given by the manufacturer [39]. All statistical analysis were conducted using ANOVAs, and subsequently, Tukey’s multiple-comparisons tests.

#### 2.9.5. Western Blot Investigation

A radioimmunoprecipitation technique was incorporated in the extraction of the lung tissue total protein. The segregation of the supernatant was achieved by centrifugation of the lung homogenates for 25 min at 10,000 rpm and 4 °C. Equal volumes of the phosphate buffer were used for the dilution of the supernatants, which were then heated to the boiling point (90–95 °C) for 10 min. The samples were then charged with 12% gels for sodium dodecyl sulfate–polyacrylamide gel electrophoresis, followed by allocation to a polyvinylidene difluoride membrane (Millipore, MA, USA) [36]. The samples were incubated with the specific antibodies at 4 °C for 12 h after being rinsed three times with Tris-buffered saline containing Tween 20 (TBST). The primary investigated antibodies included rabbit antiactin, rabbit antimyeloperoxidase (MPO), and rabbit anti-nuclear factor-κB, which were all subjected to 1:1000 dilutions (Abcam, Cambridge, UK).

### 2.10. Pharmacokinetic Study

The pharmokinetic study was conducted in accordance with the research protocol approved by the Research Ethics Committee, Faculty of Pharmacy, Port Said University (PI3156): 12 male Wistar albino rats with weights in the 220–250 g range were housed in standard polypropylene cages and were maintained under optimum laboratory conditions of free access to a standard laboratory diet and water ad libitum, temperature, humidity, and light. The rats were assigned into two groups of six. Group (I) animals were each administered an oral dose comprising 20 mg/kg of MH dispersion in PBS, as elsewhere throughout this study, and with particle sizes of 672.3 ± 32.1 and PDI of 0.576 ± 0.1. Group (II) animals were each administered an oral dose with the MH equivalent contained in the dispersion of the optimum MH-charged PCM F9 formulation [40]. Both groups were subjected to overnight fasting the day before the dose administration. Blood samples (0.50-mL) were withdrawn from the tail veins of each animal at different time intervals (0.5, 1, 2, 4, 6, 8, 12, 18, and 24 h) and were stored into EDTA-coated tubes. The plasma was segregated by centrifugation for 10 min at 3500 rpm, and each processed sample was stored at −40 °C until they were further assessed. Plasma concentrations of the residual MH were determined using the HPLC methodology previously described.

The MH pharmacokinetic parameters from both the MH-loaded PCM F9 formulation and MH suspensions were investigated for each rat utilizing noncompartmental pharmacokinetic models [31] with the Thermoscientific Kinetica^®^ software Version 5 (Cherry Street, Philadelphia, PA, USA). One-way ANOVA statistical tests were employed to differentiate between the C_max_ and AUC_0–24_ (from the maximum concentration area under the curve from 0–24 h) of both types of preparations. Furthermore, the nonparametric signed-rank test (Mann–Whitney test) was employed to appraise the difference between the medians of the T_max_ in the two treatment groups with the statistical software SPSS Version 14. A divergence at *p* < 0.05 was considered to be significant [27].

## 3. Results and Discussion

### 3.1. Experimental Design Statistical Evaluation

In order to investigate the effect of the compounding variables on the proposed responses, a 2^4^ full-factorial design was created. The construction of the 16 experimental runs and their related Q8h, EE, PS, and ZP responses are shown in Table 2. The appropriate model precision value was utilized to substantiate its suitability to maneuver the design space [41,42]. A ratio exceeding four was endorsed, which was perceived for all the dependent variables, as demonstrated in Table 3. The predicted and adjusted R2 should not be gapped with values greater than 0.20 from one another to confirm a reasonable agreement. Table 3 shows that the adjusted R2 values were consistent with the predicted R2 values for all the dependent variables [43].

### 3.2. Influence of the Compounding Variables on EE%

**Figure 1:**
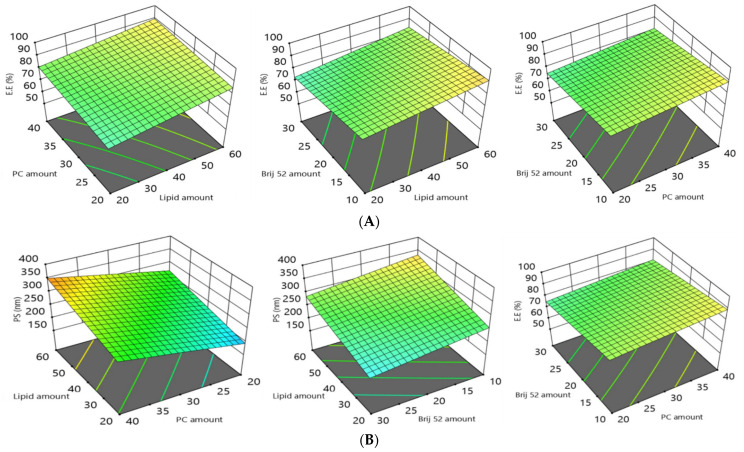

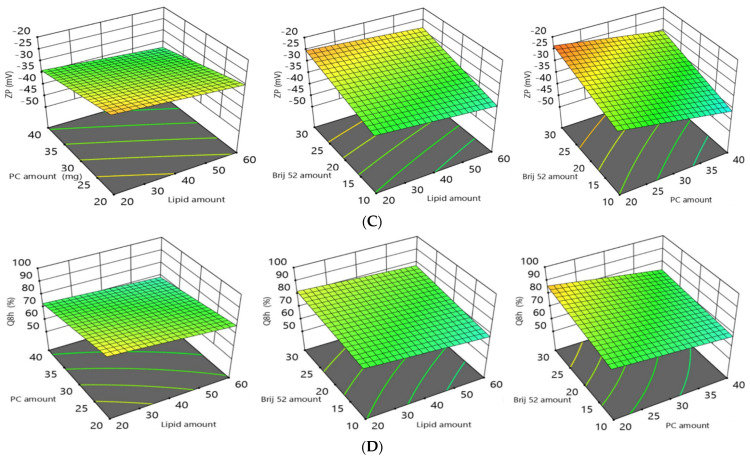
The 3D plots showing the effects of the amounts (in mg) of (*from top to bottom rows*): (**A**) lipid core type; (**B**) lipid core; (**C**) PC amount; and (**D**) Brij52 on the formulation variables: **EE%**, **PS**, **ZP**, and **Q8h** of MH-loaded PCMs. Abbreviations: MH = morin hydrate; EE% = entrapment efficiency of MH; PS = particle size; ZP = zeta potential; Q8h = % of MH release after 8h; PCMs = PEGylated chylomicrons.

The loading capability of the MH in the investigated vesicles was appraised from the entrapment efficiency percentage (EE%) estimation. The percentages of MH encapsulated in the vesicles ranged from 59.8 ± 1.3% to 98.6 ± 2.8%. ANOVA results affirmed that the model with all variables for lipid core type (Factor A), Lipid core amount (Factor B), PC amount (Factor C), and Brij52 amount (Factor D) significantly impacted (*p* = 0.0007) the EE%, as illustrated in the 3D plots (Figure 1). The variables’ impacts on the EE% can be summarized as follows. With regard to the lipid core type (Factor A), the ANOVA results indicated that the EE% of the formulations composed of compritol significantly (*p* = 0.0001) surpassed those composed of triolein; this may be attributed to the discernible lipophilicity of compritol, which is a blend of different glyceryl esters, including the diester glyceryl dibehenate, of the C-22 saturated fatty acid, behenic acid [44]. On the other hand, triolein is a symmetrical triglyceride comprising the shorter C-18 monounsaturated oleic acid esters. It is well recognized that as the hydrocarbon chain of solid lipids increase, they are predisposed to a marked decrease in the hydrophilic–lipophilic balance (HLB). The HLB is a measure of the balance of the size and strength of the hydrophilic and lipophilic moieties of a surfactant molecule and the elevated solubilizing power for hydrophobic drugs, such as MH [23]. Moreover, the unsaturation in the hydrocarbon chain in triolein predisposes it to form less tightly packed vesicles, hence increasing the drug leakage. It is worth mentioning that the manipulation of lipids that are in the form of blends of the different types of acyl glycerols (mono-, di-, and triacylglycerols) such as compritol, instead of monoacid triacylglycerols such as triolein, predisposes them to configure less-organized defected crystal lattices. These can allow for extra hollow voids for the accommodation of MH molecules, leading to a higher entrapment of the drug [45].

Increasing the amount of the lipid core (Factor B) from 20 mg to 60 mg led to a significant (*p* = 0.0002) enhancement in the entrapment of the MH, and this was in accordance with previously reported studies [45]. It was previously stated that the viscosity of the fabricated vesicular dispersion was boosted by an elevation in the amount of the lipid core [27]. The formulae incorporating elevated viscosities can impede the drug propagation to the external hydrophilic phase, accordingly conferring a higher EE% [27].

With respect to the PC (Factor C), increasing its amount from 20 to 40 mg resulted in a significant increase (*p* = 0.0025) in the EE%. The increase in PC content could predispose the assembly of a lipid core to be surrounded by PC multilayers around the lipid core, thus allowing MH to be ligated into these bilayers; furthermore, PCs promoted the compactness of the vesicles, thus hindering drug leakage [45].

The statistical investigations revealed a significant (3-fold) negative effect of the Brij amount on the EE% (*p* = 0.0004): increasing Brij from 5 mg to 15 mg predisposed the PCs to a diminished EE%. This impact was credited to the introduction of more porous voids in the vesicle bilayer, and the elevation in the amount of surfactant predisposed an increase in the configuration fluidity, thus permitting an increased drug leakage and consequential lower EE% values [46].

From the aforementioned results, it can be concluded that the formulae containing compritol as a lipid core exhibited a higher EE%; increasing the amount of lipid and the PC amount predisposed toward a higher EE%, while increasing the amount of Brij52 decreased the EE%.

### 3.3. The Polydispersity Index (PDI) and the Influence of the Compounding Variables on Particle Size (PS) Polydispersity

PDI values denote the level of monodispersity and the extent of homogeneity, with PDI values close to zero denoting monodispersity, whilst values closer to 1 denote polydispersity. The PDI values of the MH-loaded CMs shown in Table 2 ranged from 0.21 ± 0.03 to 0.58 ± 0.09. Therefore, the PDI values of the MH-loaded PCMs trended toward polydispersity, but within a convenient range [47]. Generally, the destiny of a drug’s molecules in blood circulation and the drug’s permeation across intestinal membranes are highly dependent on particle size (PS), with the smallest particles exhibiting a boosted drug permeation and an enhanced drug retention time, hence promoting its therapeutic activity. As illustrated in Table 2, **Y2**, the average particle size of the fabricated formulations of this study, ranged from 150 ± 16 to 380 ± 20 nm. The ANOVA results affirmed that the model with all four variables, (Factors A–D) had a significant impact on the PS; this is graphically displayed in the 3D plots (Figure 1). The individual variables’ impacts on the PS can be rationalized as follows.

For the lipid core type (Factor A), the PS of the formulations prepared with compritol afforded a significantly higher (*p* = 0.0046) PS than those prepared with triolein. This could be due to the higher hydrocarbon chain lengths of the fatty acid esters in compritol compared with those of the oleic acid esters in triolein; in addition, triolein’s smaller hydrocarbon chain lengths and degree of unsaturation would also predispose it to a lower EE%, thus decreasing its PS.

With respect to the lipid core amount (Factor B), increasing the amount of lipid core led to a significant increase (*p* = 0.0006) in the PS, which may have been due to the attainment of a higher viscosity of the fabricated emulsion as a consequence of the elevated amount of lipid utilized in the formulation [48].

With respect to Factor C, increasing the amount of PC led to a significant increase (*p* = 0.0004) in the PS. This finding was consistent with those findings of Aldawarsi et al. [23], who reported that increasing the phospholipid concentration increased the PS of their formulated raloxifene emulsomes. This may have been a result of the formation of multiple bilayers, which subsequently increased both the PS and the EE%. In addition, the location of a PC at the junction between the oily and aqueous phases can also enable an emulsome to encapsulate a larger amount of the target drug, resulting in larger emulsome sizes [45].

Finally, the increase in the amount of Brij (Factor D) contributed to a significant decline (*p* = 0.0041) in the PS. This impact was affirmed by the reduction in the interfacial tension of the system and the changed configuration of the produced vesicles, owing to the surface-active nature of Brij. The lower the amount of Brij, the lower its capability to support a lipid interface, hence a hindered effect on the surface tension, thus resulting in larger vesicles [20]. Correspondingly, the promoted steric fence on the surface of the vesicles associated with an increase in the amount of Brij impeded the clumping of the particles and promoted the system’s stability [49].

Based on the results, we deduced that the formulae containing compritol as a lipid core exhibited a higher PS, and increasing the amounts of lipid and PC resulted in increases in the PS, whereas increasing the amount of Brij52 decreased the PS.

### 3.4. The Influence of the Compounding Variables on Zeta Potentials (ZPs)

Zeta potentials (ZPs) can be a measure of the stability of a colloidal or vesicular system. ZP values of at least ±30 mV are considered to be required for electrostatic stabilization [50]. This implies that a sufficient electric repulsion exits between the vesicles, thus preventing their fusion and increasing their stability [46]. The estimated ZP values from the PCMs in the present study ranged from −21.6 ± 4.3 to −47.6 ± 6.1 mV (Table 2). The ANOVA results indicated that all four variables (Factors A–D) all significantly affected ZP, as graphically illustrated in the 3D plots (Figure 1). The impact of the variables on the ZP can be rationalized as follows.

A high negative charge of the emulsomes would have a positive influence on the capability of the vesicles to encapsulate the MH. Since the MH exhibited three pKa values of 4.99, 8.29, and 10.33, at a pH of 7.4 in the PBS, most of the MH molecules were electronically neutral, hence the electrostatic interaction helped in promoting the drug-loading capacity [8]. Our findings were also consistent with the findings proposed by El-Zaafarany et al. in their study [45], since negatively charged vesicles were able to totally engulf oxycarbazepine with a pKa of 10.7, which bore a positive charge.

Changing the type of lipid core (Factor A) had a significant impact (*p* = 0.049) on the ZP. This may be attributed to the lower EE% of the triolein-based formulae than that of the compritol-based ones, as mentioned previously. Thus, the MH was more shielded inside the triolein-based lipid core, so that fewer electrostatic interactions occurred between the MH and the negative charge on the surface of the vesicles, consequently resulting in a more prominent negative charge on the emulsomes [51]. Increasing the amount of lipid (Factor B) also imparted a significant effect (*p* = 0.0122) on the ZP: the greater lipid amount permitted more drug to diffuse into the solid lipid core, leading to a higher negativity on the surfaces of the emulsomes.

The ANOVA results revealed that increasing the amount of PC (Factor C) from 20 to 40 mg significantly increased (*p* = 0.0006) the negative charge imparted on the vesicle surfaces. El-Zaafarany et al. previously reported that the negatively charged surface of the emulsomes was due to the location of the negatively charged PC on the outer layer [45]. Accordingly, increasing the amounts of PC elevated the overall values of the ZPs [25]. On the contrary, increasing the amount of Brij52 (Factor D) significantly decreased (*p* = 0.0002) the overall values of the ZPs: elevation of the amounts of Brij increased the number of PEG moieties, which acted as a steric fence that could shield the surface charges, resulting in reducing the overall ZP values [27].

From the aforementioned results, we concluded that the formulae containing compritol as a lipid core exhibited higher ZPs, increasing the amount of lipid and PC amount predisposed the formulae to higher ZPs, while increasing amount of Brij52 decreased the ZPs.

### 3.5. Influence of the Compounding Variables on the Amount of Drug Released after 8 h (Q8h)

**Figure 2:**
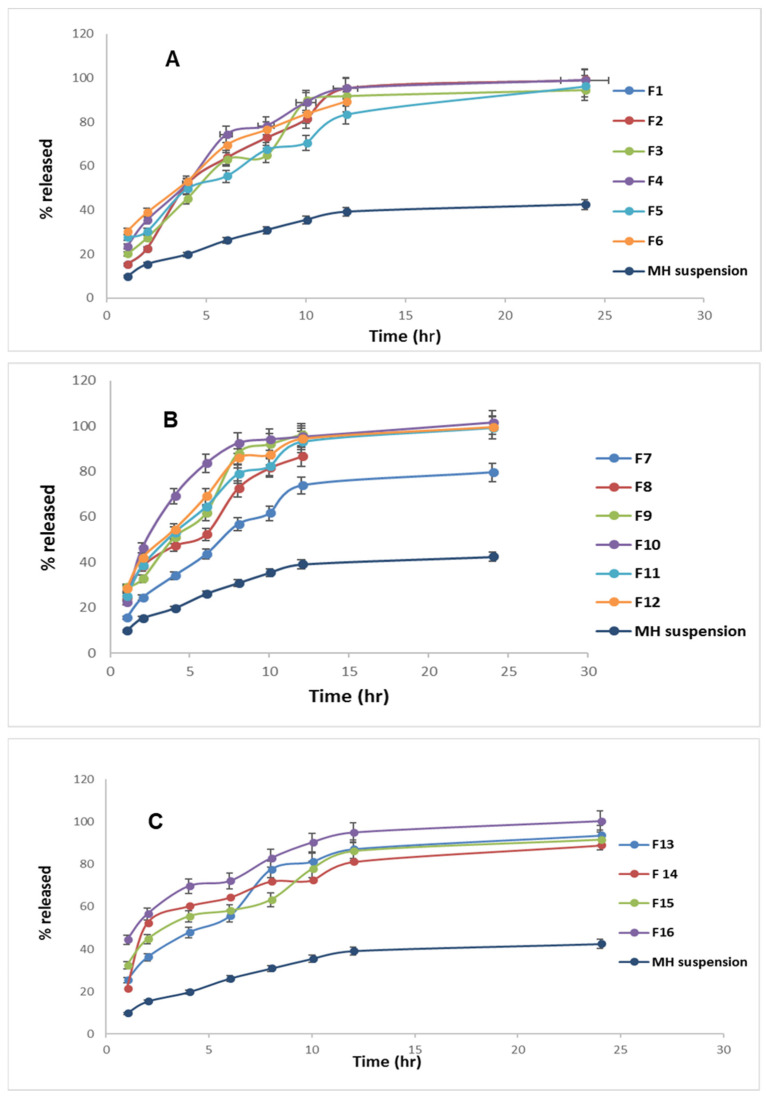
(**A**–**C**) Percentage of MH released ± S.D. from the prepared formulations (**F1**–**F16**) compared to that of the pure MH suspension.

The percentages of MH released after 8 h ranged from 56.7 ± 2.5 to 92.4 ± 3.6 (Table 2). The impact of the four variables (Factors A–D) significantly affected the Q8h, lipid core type (A), lipid core amount (B), PC amount (C), and Brij52 amount (D); these are configured as 3D response plots in Figure 1. Furthermore, Figure 2A–C demonstrate the MH release data from the tailored PCMs over the time intervals. The shape of the MH release data revealed two sequential phases: Phase I revealed a fast ejection of the drug from 18–60% during the first 2 h, and was succeeded by Phase II, which was characterized by a slower release of the drug. The higher incorporation of the drug into the lipophilic pockets in the vesicles slowed the rate of drug release, emphasizing the interlinkage between the MH EE% and its release. The ANOVA analysis of the effect of Factor A revealed that the formulations prepared using triolein exhibited a greater significant Q8h effect (*p* = 0.0026) than those prepared using compritol. This may be attributed to the effect of the more closely packed solid core surrounding the drug in the case of the compritol, hence increasing its EE% and restricting the drug release. Among other factors that can promote drug release from within a particle-encapsulated drug, the surface area:volume ratio of a particle correspondingly increases as particle size decreases [52]. On the other hand, increasing the amount of lipid (Factor B) also imparted a significant effect (*p* = 0.0315) on Q8h. Albash et al. previously reported that increasing the amount of the lipid boosted the viscosity of the fabricated vesicular dispersion, and correspondingly, drug release into the external aqueous phase was inhibited with an increasing viscosity of the formulations [27]. Increasing the amount of PC (Factor C) significantly (*p* = 0.0023) suppressed the drug release due to the formation of multiple phospholipid bilayers with increasing PC amounts [25]. On the other hand, increasing the amount of Brij52 (Factor D) significantly (*p* = 0.0057) boosted the release of MH due to the construction of less-packed, highly porous bilayers with increasing amounts of Brij52 [27].

Based on these results, we deduced that the formulae containing compritol as a lipid core exhibited lower drug release rates, and hence lower Q8h values; increasing amounts of lipid and PC tended to lower the Q8h values, while increasing amounts of Brij52 increased the drug release, and consequently, the Q8h.

### 3.6. Selection and Validation of the Optimal MH-Loaded PCM Formulation

The optimal formulation chosen for the in vitro investigations (F9) was based upon the analysis of the effects of the dependent variables using Design Expert. Formulation F9 had a computed desirability value of 0.784, and was therefore constituted using 20.0 mg of compritol as the solid lipid for the lipid core, 20.0 mg of PC, and 30.0 mg of Brij52. The validities of the models tested were affirmed by estimating the percentage differences between the predicted and observed values of the four factors considered; i.e., %EE, PS, ZP, and Q8h. As shown in Table 3, the low (less than 10%) discrepancy percentage as an absolute value supported the appropriateness of the statistical design in the data analysis [53].

### 3.7. In Vitro Investigations of the Optimum MH-Loaded PCM Formula

#### 3.7.1. Differential Scanning Calorimetry (DSC)

**Figure 3:**
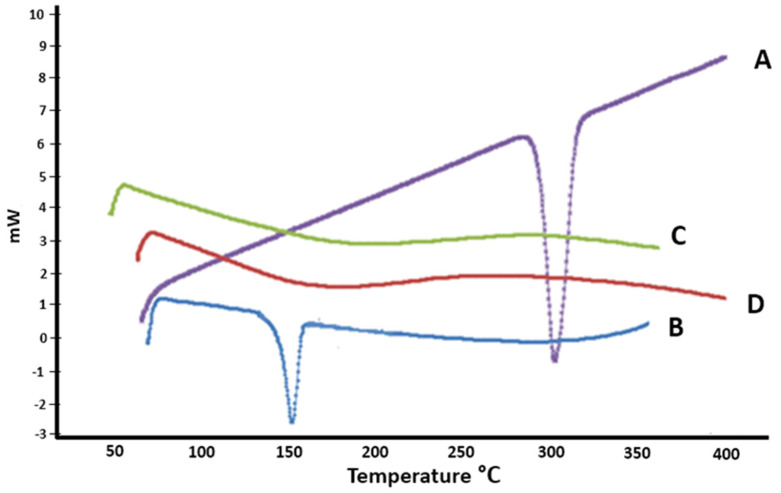
DSC thermograms: A: pure MH; B: cholesterol; C: F9 formulation alone; and D: the optimum MH-loaded formulation F9.

The DSC thermograms of pure MH, plain lyophilized formula, and cholesterol, in conjunction with the optimum lyophilized MH-loaded PCMs (formulation F9), are shown in Figure 3. The thermogram of the pure MH revealed a clear sharp endotherm at approximately 295.4 °C that was related to its melting point [9]. For cholesterol, the endothermic peak was at 148.5 °C, corresponding to its melting point [54]. However, the thermograms of F9 and of the lyophilized plain formula revealed no distinctive peaks corresponding to that of the MH. This suggested that not only the MH, but also the other components (cholesterol, Brij52, and compritol) formed an amorphous instead of a crystalline configuration, resulting in the optimum loading and accommodation of the drug in the vesicles.

#### 3.7.2. Transmission Electron Microscope TEM

**Figure 4:**
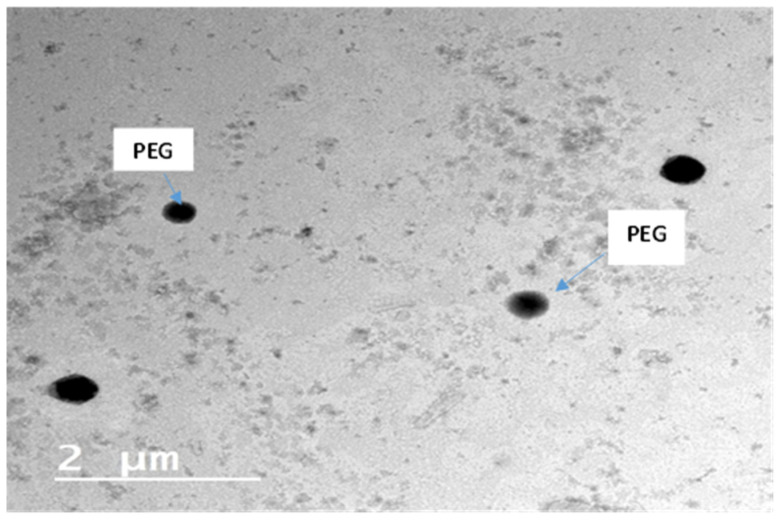
TEM of the elected optimized PEGylated chylomicron (F9).

The TEM image (Figure 4) affirmed the spherical shape of the vesicles, with no vesicles having obvious irregular shapes. Furthermore, the surfaces of the vesicles were smooth, and the vicinity of the PEG at the edges of the vesicles could be inferred from the TEM image [54]. The complete transformation of the drug into an amorphous configuration was confirmed by the absence of any drug crystals, which was in agreement with the DSC results.

#### 3.7.3. Effect of Storage on the In Vitro Characteristics of the Optimal Formula

The impact of short-term storage on the optimum MH-loaded formulation F9 for 3 months at 4 °C and at 25 °C indicated that no significant (*p* > 0.05) changes to the investigated parameters of EE%, PS, and Q8h could be discerned (Supplementary Materials Table S1). This may be ascribed to the PEGylated surfactant-induced steric stabilization [54]. The beneficial role of the PEG coating in increasing the stability of the prepared vesicles also was noted.

### 3.8. Ex Vivo Morin Hydrate (MH) Gut-Permeation Study

**Figure 5:**
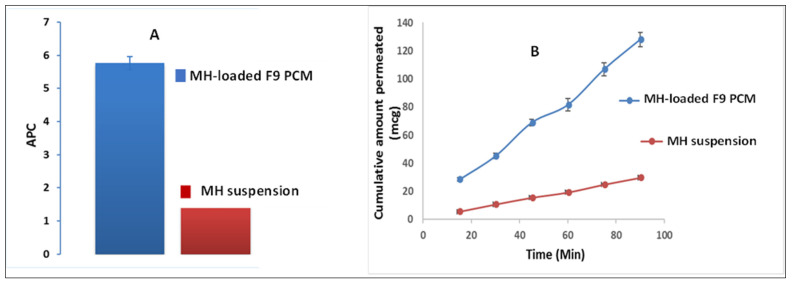
MH ex vivo gut-permeation study showing: (**A**) apparent permeability coefficient (APC) ± S.D. of MH-loaded formulation F9 PCM versus MH suspension; and (**B**) cumulative amount of MH permeated (μg) ± S.D of MH-loaded F9 PCM versus the pure MH suspension time profile.

A comparative estimation of MH intestinal lavage via the non-everted gut sac technique was conducted with the pure MH suspension versus the optimum MH-loaded formulation F9. This was conducted as a trial to imitate the planned in vivo study (vide infra) in order to anticipate the kinetic and biological availability of the drug. After 90 min, the optimized PEGylated chylomicron formulation F9 developed a superior flux (J*_max_* = 1.4 ± 0.063 μg/cm^2^/min) relative to that of the pure MH suspension (J*_max_* = 0.37 ± 0.018 μg/cm^2^/min). The apparent permeability coefficient (APC) of the pure MH suspension was 1.52 × 10^−4^ cm/min, while that of the MH-loaded formulation F9 was 5.76 × 10^−4^ cm/min, indicating a significant distinguishing (*p* < 0.05) ~4-fold increment in the permeation of the F9 over the pure MH suspension (Figure 5A). The superiority of the F9 over the pure MH suspension during the entire experimental time can be seen in Figure 5B. Moreover, this denoted to a great extent that the system could successfully bypass the obstacles that hindered the drug absorption [55,56].

### 3.9. Investigation of In Vitro Anti-MERS-CoV Activity

#### 3.9.1. MTT Cytotoxicity Assay

**Figure 6:**
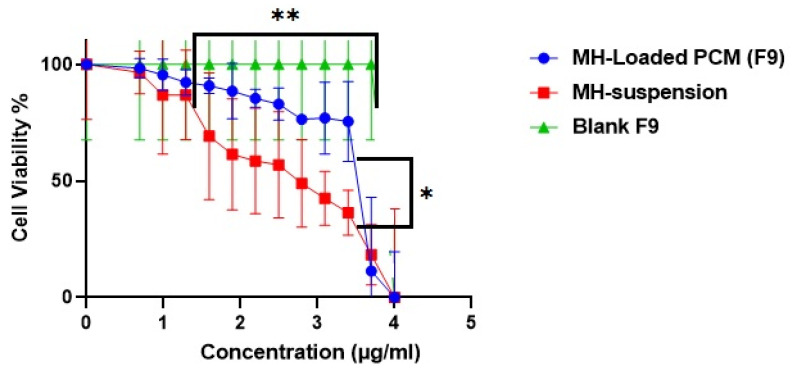
In vitro cytotoxicity of MH-loaded PCM (F9) (blue); B: MH suspension (red); and C: blank (F9) (green). * Denotes significance level at *p* < 0.05, and ** denotes significance level at *p* < 0.01.

Ideally, an antiviral’s activity occur at a minute concentration, and be cytotoxic to an extent at higher concentrations. The concentration for 50% cell cytotoxicity (TC50) of the host cells with the F9 MH formulation’s best fit was computed to be 5.27 µg/mL, compared with the corresponding value of 2.53 µg/mL for the pure MH suspension (Figure 6). No cytotoxicity could be discerned for the MH-free F9 formulation when tested up to a concentration >18.4 µg/mL. The safety margin of the F9 MH formulation was significantly (*p* < 0.05) superior to that of the MH suspension. The TC50 investigation was confirmed not only when tailoring the serial dilutions used in further investigations within the safety range, but also confirmed the capability of the PCMs to guarantee a high protection level in the host cells throughout the treatment course.

#### 3.9.2. Plaque Assays

**Figure 7:**
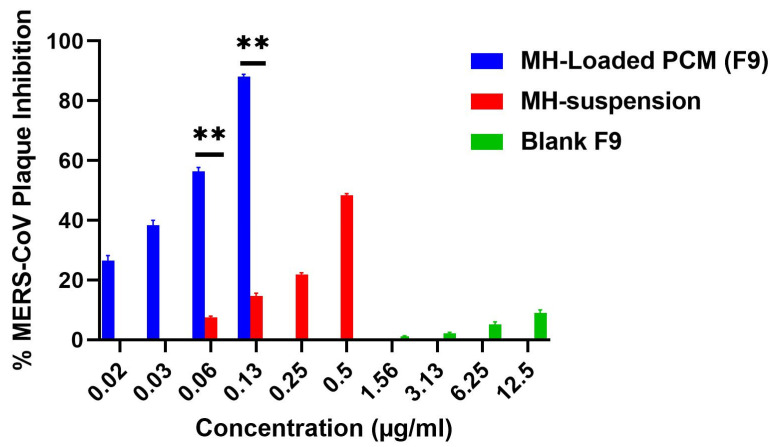
MERS-CoV plaque assays ± S.D. *Left to right*: (*blue*) F9; (*red*) pure morin hydrate (MH) suspension; (*green*) MH-free F9. ** Denotes significance level at *p* < 0.01.

The results of the viral plaque assays are depicted in Figure 7. The inhibition efficacy of the MH suspension was 48% ± 2.3% at a concentration of 0.125 µg/mL versus 88% ± 3.4% for the MH-loaded F9 formulation at the same concentration. In contrast, the MH-free blank F9 formulation inhibited the formed viral plaques by only 9% at a concentration of 12.5 µg/mL. MH therefore possessed antiviral activity even at minute concentrations. The antiviral activity of F9 (EC_50_ = 0.073 µg/mL) against the Vero E6 cells infected with MERS-CoV was significantly superior (*p* < 0.01) to that of the MH suspension (EC_50_ = 0.13 µg/mL). The capability of the fabricated vesicles to conjugate either by endocytosis or by fusion to the viral cells may have accounted for the enhanced antiviral activity of the MH in its F9 formulation.

The attachment of the PEGylated vesicles to the viral cellular membranes increased the concentration and consequent enhancement of the thermodynamic activity gradient of the MH, thus promoting the permeation ability of lipophilic moieties such as the vesicle-encapsulated MH. Furthermore, the compounding ingredients of the CMs played a crucial role in improving the antiviral activity, since the lipids and surfactant incorporated in the formulation enhanced the diffusion of the MH through the viral cells [57]. El-Halim et al. also demonstrated the vital role of nanovesicular proniosomes in improving the antiviral activity of curcumin against the herpes simplex virus [58].

The relative effectiveness of a drug in prohibiting viral multiplication versus its cytopathic effect is given by a selectivity index (SI) computed by dividing the concentration at 50% cellular cytotoxicity by the 50% effective concentration; i.e., SI = CC50/EC50 (39). Using this measure, the F9’s SI value of 72.3 was significantly superior (*p* < 0.01) to that of the MH suspension (19.4), confirming the greater selectivity achieved by the F9 formulation.

### 3.10. Computational Analysis

#### 3.10.1. Molecular Modeling Study

Jo et al. [59] demonstrated that several flavonoids produced their antiviral activities by effectively reducing the proteolytic activity of the MERS-CoV chymotrypsin-like protease (MERS-CoV 3CLpro). The molecular docking study reported herein therefore was conducted with this protease to rationalize the antiviral activity of the MH. As described above in Section 2.8, Discovery Studio was used to conduct the docking study. CDOCKER was used to generate the docking poses based on the superiority of their scores and their efficient binding interlinkages. The CDOCKER binding energy scores, as well as the evolution of H-bonds and/or hydrophobic contacts with the catalytic Cys148-His41 dyad within the functioning location of the S1 binding site [33], were the determinants able to influence the binding aptitude to the binding cavities of MERS-CoV 3CL^pro^.

**Figure 8:**
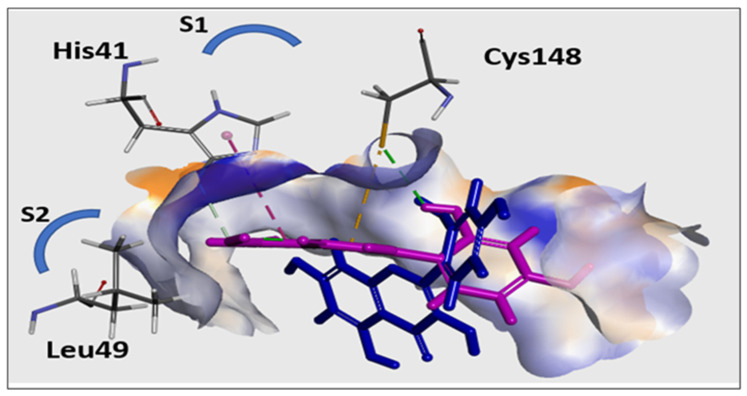
The two different binding patterns of MH to the active site of MERS-CoV 3CL^pro^: Binding mode A (*lower blue molecular structure*); and Binding mode B (*upper magenta molecular structure*). In Binding mode B, MH interacted with the catalytic Cys148-His41 dyad within the active site of the S1 binding site.

**Figure 9:**
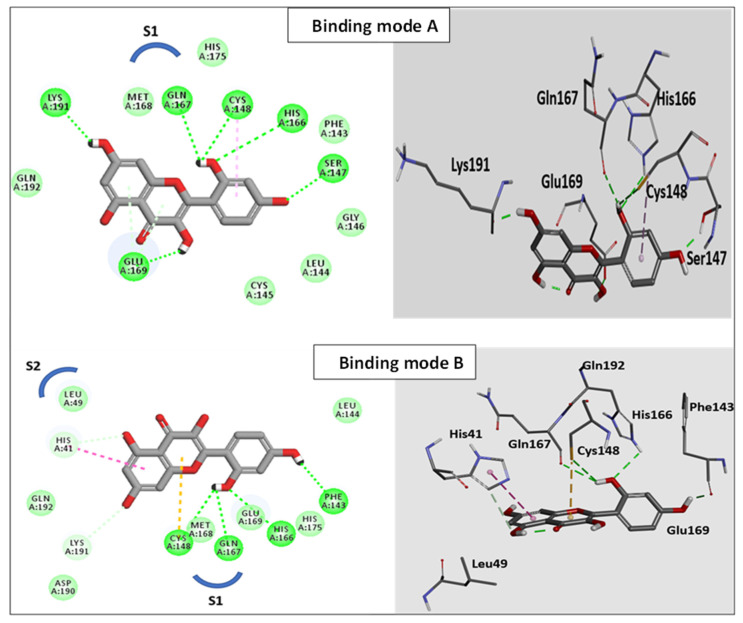
The 3D structural models of morin (Binding mode A and Binding mode B) on the active site of MERS-CoV 3CL^pro^ showing a two-dimensional representation of the interacting pattern of morin with 3CLpro enzyme. The H-bond interactions are represented by dotted lines.

Redocking of the co-crystallized inhibitor into the binding site of 3CL^pro^ was used to validate our in silico docking results. The alignment depicted excellent coexistence between the X-ray conformer and the best-fitted pose **A** with RMSD = 0.56 A°, indicating that the CDOCKER docking protocol effectively fetched valid docking poses. The obtained docking poses of the MH occupied the S1 and S2 binding sites, with scoring energies of −27.1 to −28.9 kcal/mol with different binding patterns (Figure 8). In binding mode A (Figure 9), the ligand was perfectly positioned in the S1 binding site, where a network of six H-bonds (to Ser147, His166, Gln167, Glu169, and Lys191) were mapped, which stabilized the MH on the active site of the enzyme. Additionally, a critical hydrogen bond was configured with the catalytic Cys148.

In Binding mode B, the MH occupied the S1 site and extended to the S2 pocket of the enzyme. In the docked pose B, the ligand was aligned with the catalytic Cys148–His41 dyad within the functioning spot, where the 2-hydroxyphenyl formed a cluster of H-bonds with Cys148, His166, and Gln167. Moreover, the phenyl ring of the chromen-4-one scaffold formed π–π stacking with His43. In addition, the chromen-4-one fragment formed hydrophobic contact with the Leu49 on the S2 binding site. In conclusion, the docking study indicated that the MH was oriented toward the catalytic Cys148–His41 dyad within the active site of the MERS-CoV 3CLpro. This study strongly suggested that the potent antiviral inhibitor activity of MH could be explained by its minimizing of the proteolytic activity of MERS-CoV.

#### 3.10.2. In Silico Predictive Computer-Aided Absorption, Distribution, Metabolism, Elimination, and Toxicity (ADME) Screening for MH

**Figure 10:**
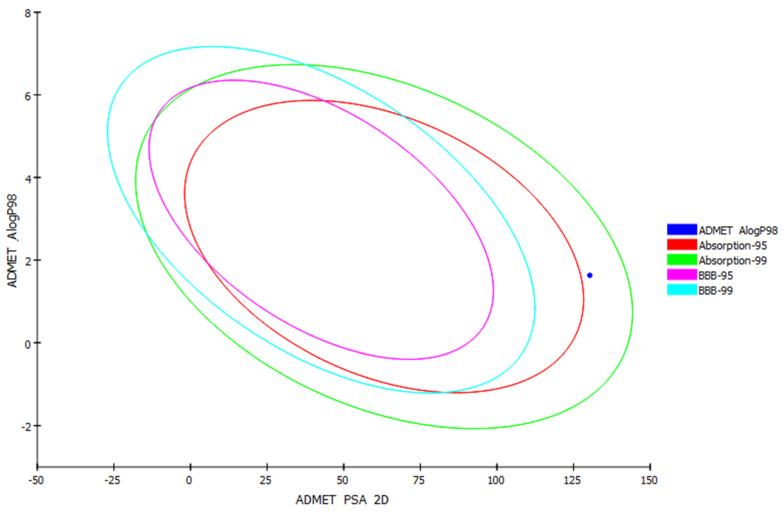
Blood–brain barrier (BBB) plot and human intestinal absorption (HIA) for MH.

Computer-aided absorption, distribution, metabolism, elimination, and toxicity (ADME) studies were conducted in order to evaluate the pharmacokinetic properties of the MH. These studies were correlated with the chemical structure of the MH, in which the following parameters are analyzed: *(a)* blood–brain barrier level (BBB Lev); *(b)* absorption level; *(c)* aqueous solubility level (AQ SOL Lev); *(d)* hepatotoxicity; *(e)* cytochrome P450 2D6 (CYP2D6 Prob) inhibition probability; *(f)* probability of cytochrome P450 (CYP 2D6); *(g)* 2D polar surface area atom-based Log P98 (AlogP 98); and *(h)* ADMET PSA Lev. The results obtained are depicted as an ADMET plot (Figure 10) using the calculated PSA 2D and Alog P98 properties.

BBB and human intestinal absorption (HIA) plots were also determined for the MH. In the BBB plot, the MH fell outside the 99% and 95% ellipses, indicating that it may not have been ready to penetrate the blood–brain barrier. Consequently, MH may be anticipated to possess low CNS side effects. In the HIA plot, the MH was located inside the 95–99% ellipses, and thus it was predicted to have low intestinal absorption. Significantly, the aqueous solubility level was expected to be 2, indicating low aqueous solubility. The CYP2D6 value predicted the inhibitory and non-inhibitory aspect of the MH on the cytochrome P450 2D6 enzyme. The MH was predicted to be a noninhibitor of CYP2D6, so drug–drug interactions and side effects such as liver dysfunction were not expected upon administration. In general, molecules with PSA > 140 have a poor bioavailability. The bioavailability radar (SwissADME [60]) indicated that the MH was presumed to possess a low oral bioavailability, with a PSA = 140. The ADMET parameters are presented in Table S2 in the Supplementary Materials.

### 3.11. In Vivo Study

A comparative in vivo study with a MERS-CoV-challenged mice model was conducted to test the differences between the impacts of the pure MH suspension and the MH-loaded PCM formulation F9 on resolving the viral infection and its consequences in the animal model. Fundamentally, sequential harsh pathological changes encompassing lymphoid infiltration and accompanying interstitial inflammation can be detected following infection with MERS-CoV [37]. Additionally, there is oxidative stress resulting from the depletion of the overall concentration of thiol content along with GSH, CAT, and SOD functionality. In addition, it can be noted that the lung lavage levels of IFN-γ, TNF-α, and IL-6 will be massively increased, whereas the level of IL-4 will be diminished, in this infection. Furthermore, many clinical studies have illustrated that the disease harshness and lethality for SARS patients was highly integrated with the upregulation of proinflammatory cytokines/chemokines, such as IFN-γ, IL-8, and IL-6. It is also noteworthy that the massive production of ILs and IFN-γ throughout the sequel of the infection points to the induction of an enormous antiviral response associated with MERS-CoV infection. The induction of both IL-6 and TNF therefore can be considered as potent manifestations of severe respiratory viral infection from MERS and SARS [39]. Therefore, in the study reported herein, the antiviral efficacy of the MH, along with its anti-inflammatory and antioxidant activities to suppress the harsh MERS-CoV infection and its consequences, were affirmed through the assessment of the levels of IL-6, IL-4, TNF-α, IFN-γ, and SOD in the BALF.

#### 3.11.1. Viral Titer Investigation in Acutely Infected Lungs by Adopting a Plaque Assay

**Figure 11:**
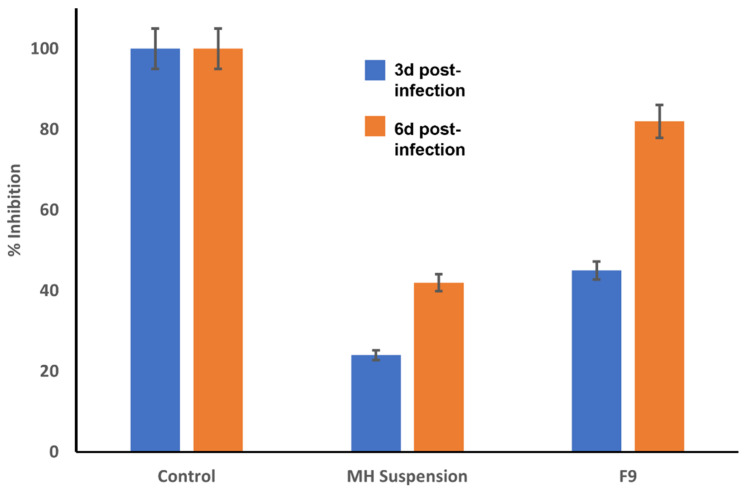
MERS-CoV titer via plaque assay (pfu/g lung homogenate ± S.D) at day 3 and day 6 postinfection after treatment with either MH-loaded formulation F9 or pure MH suspension.

In accordance with previous studies, after 6 h of viral infection, the virus could be well discerned in the respiratory tracts of the C57BL/6 mice, whereas 7 days postinfection, the virus could scarcely be monitored [1]. Therefore, the experiment was not conducted beyond 6 days postinfection. As illustrated in Figure 11, the % plaque prohibition values from the postinfection F9-treated mice after 3 and 6 d were 51% and 82%, respectively, and were significantly elevated relative to those from the postinfection MH-suspension-treated mice, at 27.1% and 44.8%, respectively.

#### 3.11.2. Assessment of IL-4, IL-6, TNF-α, IFN-γ, and Superoxide Dismutase (SOD) in Bronchial Alveolar Lavage Fluid (BALF)

The IFN-γ level in the BALF was elevated to 122 ± 14.3 pg/mg protein postinfection in comparison with that of the control group; i.e., 43.5 ± 5.60 pg/mg protein. The levels of TNF-α and IL-6 in the control group were 44.8 ± 9.40 and 22.2 ± 7.60 pg/mg protein, respectively, which were also significantly (*p* < 0.05) increased to 89.3 ± 8.8 and 188 ± 21.3 pg/mg protein, respectively. All of the noted massive increases in these levels signified the evolution of the inflammatory storm. In contrast, the levels after exposure to MH were significantly different. The levels of IFN-γ after the administration of either the MH suspension or the MH-loaded formulation F9 were reduced to 82.7 ± 7.51 and 54.3 ± 6.30 pg/mg protein, respectively. In addition, it can be seen that these IFN-γ levels were also significantly (*p* < 0.05) different from each other. The TNF-α levels were also significantly (*p* < 0.05) reduced to 33.6 ± 4.4 pg/mg protein after the administration of the MH-loaded formulation F9 and to 57.4 ± 6.2 pg/mg protein after the administration of the pure MH suspension. The levels of IL-6 declined to 65.6 ± 7.40 pg/mg protein for the MH-loaded formulation-F9-treated mice, and to 112.9 ± 14.6 pg/mg protein for the pure MH suspension-treated mice.

The ANOVA results for all of these results confirmed that the MH-loaded formulation F9 significantly (*p* < 0.001) suppressed the IFN-γ, IL-6 and TNF-α levels more than those in both the untreated group and the group that received the pure MH suspension.

**Figure 12:**
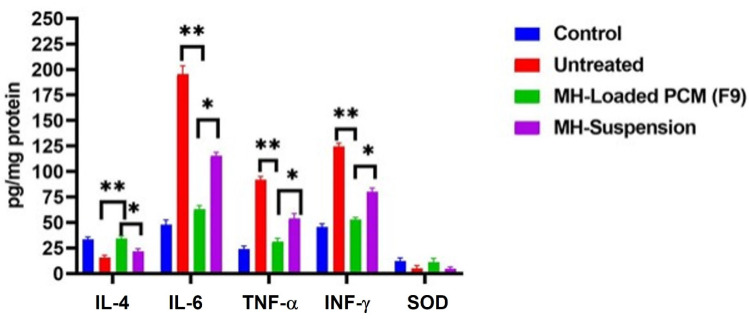
Bronchial alveolar lavage fluid levels of INF-γ, TNF-α, IL-4, and SOD ± S.D in the experimental groups: control, untreated, infected group with the pure MH suspension treatment, and infected group with the MH-loaded formulation F9 treatment. * Denotes significance at *p* < 0.05; ** denotes significance at *p* < 0.01.

Ultimately, MERS-CoV infection led to massive inflammation that was characterized by the suppression of IL-4 levels in the BALF to 13.7 ± 3.1 pg/mg protein, compared with the levels of 35.2 ± 4.7 pg/mg protein in the control group. On the other hand, after MH treatments, there were significant (*p* < 0.001) upregulations in the IL-4 levels to 32.7 ± 3.60 pg/mg protein in the F9-treated mice and to 23.5 ± 2.10 pg/mg protein in those treated with the MH suspension. Furthermore, the levels of SOD were 3.1 ± 0.51 and 0.23 ± 0.008 pg/mg protein in the control and viral infected groups, respectively, whereas these levels were elevated to 0.92 ± 0.12 and 2.2 ± 0.35 U/mg protein in the cases of the groups treated with the F9 and MH suspensions, respectively. The results summarized in Figure 12 were in accordance with other findings reported previously in various related studies [39,61].

Based on these findings, the MH-loaded formulation F9 successfully inhibited plaque formation at days 3 and 6 postinfection, and suppressed the levels of IFN-γ, TNF-α, and IL-6 in the BALF to a greater extent than that by the pure MH dispersion alone. In addition, the MH-loaded formulation F9 significantly increased the levels of SOD and Il-4 to a greater extent than did the pure MH dispersion.

#### 3.11.3. Histopathological Investigations

**Figure 13:**
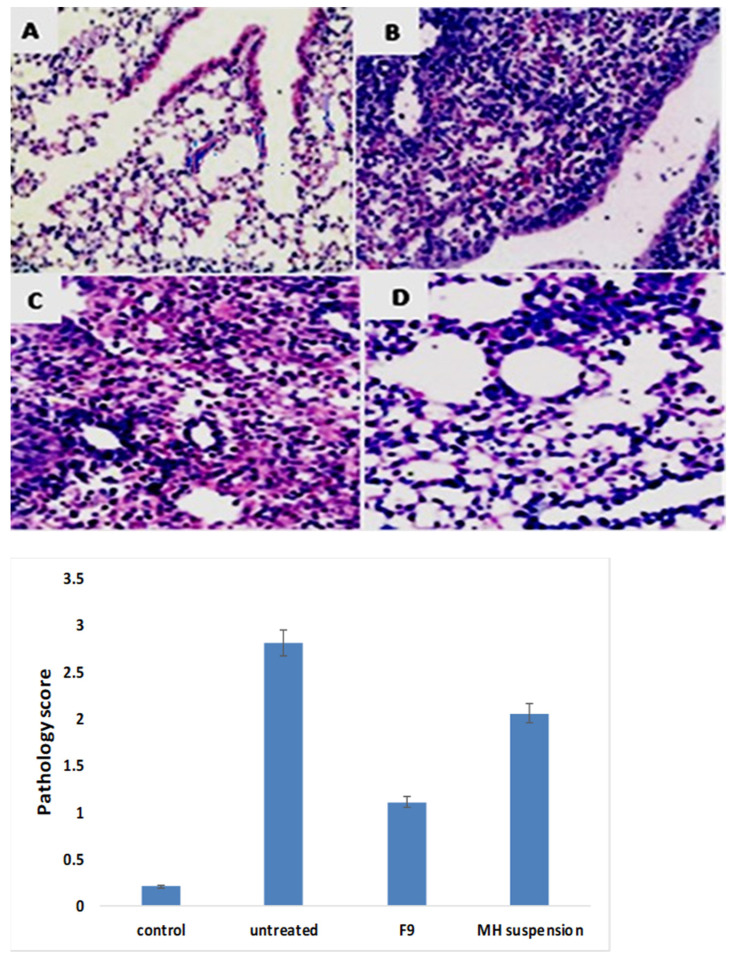
*Top*: lung histopathology (H&E staining; 400X magnification; Scale: bar = 50 μm) of: (**A**) control group; (**B**) untreated viral infected group; (**C**) group treated with pure MH suspension; and (**D**) group treated with MH-loaded formulation F9. *Bottom*: pathology score histogram.

The histopathologies of lung tissues acquired from the different groups are shown in Figure 13A–D. The lung histology of the control group in A showed no characteristic pathological alteration in the specimens. In contrast to that of the control group, the untreated virally infected group (B) revealed prominent pathological alterations manifested by significant cellular aggregation and infiltration with extensive fibrin deposition on the alveolar septae, predisposing the tissue to lung edema. The administration of MH caused a marked suppression in all of those alterations, as can be seen in C (treatment with pure MH suspension) and D (treatment with MH-loaded formulation F9). Furthermore, the treatment with the latter significantly diminished all of these changes to a greater extent than did the pure MH suspension. The histological scores of the different groups are displayed in the histogram.

#### 3.11.4. Western Blot Investigation

**Figure 14:**
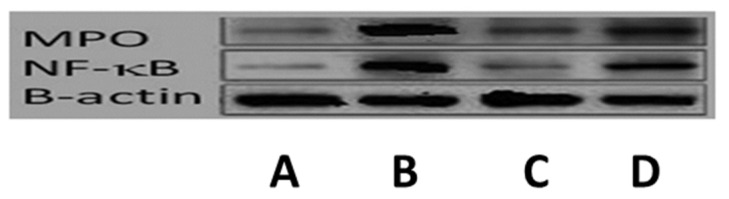
Western blotting for detection of myeloperoxidase (MPO) and NF-κB in: A, control group; B, untreated viral infected group; C, group treated with MH-loaded formulation F9; and D, group treated with pure MH suspension.

Myeloperoxidase (MPO) level assessments indicated the extent of neutrophil agglomeration in the lung tissue; furthermore, NF-κB played a crucial role in domination of the inflammatory mediators. In the present study, the expressions of MPO and NF-κB were assessed using a Western blot investigation. As shown in Figure 14, MERS-CoV infection significantly elevated MPO and NF-κB expression compared with the control group. Treatment with MH markedly suppressed the protein expression compared with the infected group, with the administration of the MH-loaded formulation F9 significantly diminishing their expression compared with their expression in the group treated with the pure MH suspension and the virally infected untreated group.

### 3.12. Pharmacokinetic Study

**Figure 15:**
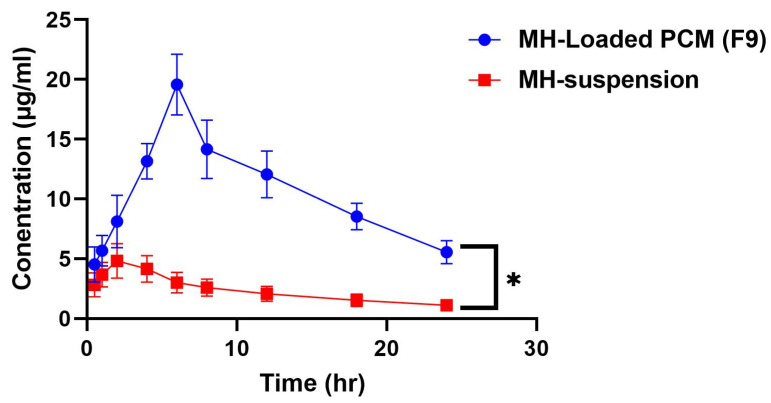
Concentration profiles of morin hydrate versus time ± S.D. after oral administration of MH-loaded formulation F9 and pure MH dispersion. * Denotes significance level at *p* < 0.05.

The plasma concentration-time profiles for both the pure MH dispersion and the MH-loaded formulation F9 are shown in Figure 15. From the pharmacokinetic parameters summarized in Table 4, it can be seen that the parameters of the MH-loaded formulation F9 were significantly (*p* < 0.05) greater than those of the pure MH dispersion. The C_max_ of the MH-loaded formulation F9 was 19.6 ± 2.60 µg/mL, and was significantly (*p* < 0.05) higher than that of the pure MH dispersion (4.82 ± 0.84 μg/mL). The corresponding T_max_ values at 6 h were 3-fold higher for the MH-loaded formulation F9, likely due to lengthened dislodging of the MH from the vesicles. Furthermore, the corresponding AUC_0–24_ was computed to be 253.1 ± 33.6 µg·h/mL, and was remarkably significantly (*p* < 0.05) higher than the AUC_0–24_ of the pure MH suspension (56.1 ± 16.2 µg·h/mL /mL). The boosted invasion of MH across the gastrointestinal tract (GIT) segments accounted for the significant 4.5-fold elevation in the relative bioavailability of the MH-loaded formulation F9 compared to the drug dispersion alone. In addition, the high relative bioavailability of the MH-loaded formulation F9 compared to that of the pure MH suspension (450%) confirmed that the composition of the former was the key to the improvement in the oral bioavailability of the MH and to extending its biological efficacy. The incorporation of PEG may therefore have offered enhanced permanence levels in the bloodstream, hindering interference with plasma opsonins and thus expanding the drug circulation period in the blood to sustain its therapeutic impact.

Thus, from the ex vivo and pharmacokinetic data, we deduced that the MH-loaded PCMs could overcome obstacles facing MH alone. These obstacles included low μg/mL solubility and permeation, which were predicted from the in silico ADME screening of the MH. Our findings emphasized the significant antiviral activity of MH and the essential blocking by MH of the evolved cytokines and free radicals accompanying the oxidative and inflammatory damage caused by the MERS-CoV infection. The results of the study clearly confirmed the boosted antiviral efficacy of the MH-charged CMs relative to that of only a pure MH suspension. As a result of the findings reported herein, MH-charged CMs can be successfully included in either the prophylaxis or eradication of the drastic oxidative stress and inflammation initiated by infection by MERS-CoV, and potentially also by COVID-19, which warrants further studies.

## 4. Conclusions

In this study, PEGylated chylomicrons were targeted for utilization as prospective nanocarriers for the oral delivery of morin hydrate. Sixteen formulae were tailored using a 2^4^ full-factorial design. Formulation F9 was selected as the optimal formulation based upon the results for the ZP, EE%, Q8h, and PS factors. Formulation F9 exhibited the highest EE%, and had the smallest PS and restricted release of the drug. Formulation F9 was confirmed to possess an improved drug-permeation ability over a pure MH suspension, according to the results of the ex vivo and pharmacokinetic studies. Regarding concerns with the safety and efficacy of an antiviral, formulation F9 proved to be superior to the pure MH suspension. Furthermore, formulation F9 displayed a vital role in the suppression of MERS-CoV-induced inflammation, along with its outstanding antioxidant activity, and was clearly superior to the pure MH suspension in its antiviral, antioxidant, and anti-inflammatory activities. The molecular modeling study indicated that the MH is perfectly positioned with respect to the Cys148–His41 catalytic dyad within the functioning post of the 3CL protease. Consequently, MH had a high aptitude to suppress the MERS-CoV 3CL^pro^ enzyme. Ultimately, the data obtained and reported herein confirmed the potential of formulation F9 to be used as a promising delivery medium for MH orally, either in prophylaxis or when resolving exaggerated MERS-CoV (and potentially COVID 19) respiratory infections.

## Figures and Tables

**Table 1:** 2^4^ Full-factorial design parameters manipulated to optimize the morin-hydrate-loaded polyethyleneglycolated chylomicrons (PCMs) and the adopted levels of independent variables.

**Factors (Independent Variables)**	**Levels of Variables**	
	***Low***	***High***
A: Lipid core type	**Compritol**	**Triolein**
B: Lipid core amount	20 mg	60 mg
C: PC amount	20 mg	40 mg
D: Brij52 amount	10 mg	30 mg
**Response (Dependent variable)**	**Desirability constraints**	
Y1: EE%	Maximize	
Y2: PS	Minimize	
Y3: ZP	Maximize (as an absolute value)	
Y4: Q8h	Maximize	

Abbreviations: **EE%** = entrapment efficiency%; **PS** = particle size; **ZP** = zeta potential; **Q8h** = % of MH released after 8 h; **PC** = phosphatidylcholine.

**Table 2:** 2^4^ Full-factorial experimental design: experimental runs, independent variables, and estimated responses of morin-hydrate-loaded PCMs.

Formula	A (Lipid Core Type)	B (Lipid Core Amount) g	C (PC Amount) g	D (Brij52) Amount g	Y1 (EE%)	Y2 (PS)	Y3 (ZP)	Y4 (Q8h)	PDI
**F1**	Compritol	20	20	10	82.8 ± 1.9	227.3 ± 11.3	32.4 ± 4.8	72.8 ± 3.5	0.25 ± 0.02
**F2**	Triolein	20	20	10	73.4 ± 1.2	203.3 ± 14.7	28.1 ± 3.1	84.7 ± 4.7	0.21 ± 0.03
**F3**	Compritol	60	20	10	93.8 ± 3.4	294.5 ± 17.2	35.3 ± 7.2	64.7 ± 4.1	0.31 ± 0.07
**F4**	Triolein	60	20	10	84.7 ± 4.1	247.7 ± 13.3	33.4 ± 6.4	78.2 ± 6.3	0.34 ± 0.05
**F5**	Compritol	20	40	10	89.4 ± 5.2	278.8 ± 22.4	42.6 ± 8.9	67.3 ± 5.5	0.28 ± 0.085
**F6**	Triolein	20	40	10	81.2 ± 2.3	255.2 ± 28.6	34.6 ± 5.1	76.3 ± 3.9	0.32 ± 0.091
**F7**	Compritol	60	40	10	98.6 ± 2.8	378.1 ± 19.6	47.6 ± 6.1	56.7 ± 2.5	0.48 ± 0.086
**F8**	Triolein	60	40	10	87.8 ± 1.9	287.8 ± 24.3	39.1 ± 5.8	72.3 ± 6.7	0.39 ± 0.074
**F9**	Compritol	20	20	30	79.1 ± 2.2	177.3 ± 18.9	21.6 ± 4.3	87.7 ± 9.2	0.28 ± 0.042
**F10**	Triolein	20	20	30	59.8 ± 1.3	154.3 ± 16.3	21.7 ± 2.7	92.4 ± 3.6	0.26 ± 0.031
**F11**	Compritol	60	20	30	89.7 ± 2.9	227.9 ± 20.4	26.3 ± 3.2	78.8 ± 4.4	0.47 ± 0.10
**F12**	Triolein	60	20	30	72.2 ± 1.1	221.9 ± 22.8	24.6 ± 3.9	85.7 ± 7.1	0.44 ± 0.088
**F13**	Compritol	20	40	30	81.5 ± 1.7	247.8 ± 21.2	28.1 ± 4.7	77.5 ± 4.4	0.42 ± 0.067
**F14**	Triolein	20	40	30	71.8 ± 3.5	233.6 ± 19.3	29.7 ± 3.3	71.9 ± 5.3	0.39 ± 0.034
**F15**	Compritol	60	40	30	91.3 ± 6.7	337.3 ± 29.6	30.1 ± 2.8	63.2 ± 3.3	0.58 ± 0.090
**F16**	Triolein	60	40	30	81.4 ± 3.7	259.4 ± 22.7	32.5 ± 5.7	82.8 ± 3.8	0.52 ± 0.11

Notes: All values are reported as mean ± SD (*n* = 3); cholesterol amount was constant in all formulae (10.0 mg). Abbreviations: **Y1** = entrapment efficiency percentage (**EE%**); **Y2** = particle size (**PS**); **Y3** = zeta potential (**ZP**); **Y4** = **Q8h** percentage of MH released after 8 h; **PDI** = polydispersity index.

**Table 3:** 2^4^-Output factorial analysis data of the MH-loaded PEGylated chylomicron (PCM) formulae and the predicted observed responses of the optimal F9 formulation.

Responses	EE (%)	PS (nm)	ZP (mV)	Q8h (%)
R^2^	0.9842	0.9739	0.9748	0.814
Adjusted R^2^	0.9526	0.9216	0.9245	0.746
Predicted R^2^	0.8382	0.7825	0.7423	0.61
Adequate precision	20.333	15.583	15.234	12.2
Significant factors	A, B, C, D	A, B, C, D	A, B, C, D	A, B, C, D
Observed value of the optimal formulation (F9)	79.1	177.3	−21.6	87.7
Predicted value of the optimal formulation (F9)	77.3	167.2	−21.36	83.1
Absolute deviation %	2.2	5.67	1.38	5.25

Abbreviations: **EE%** = entrapment efficiency percentage; **PS** = particle size; **ZP** = zeta potential; **Q8h** = % of MH released after 8 h.

**Table 4:** Pharmacokinetic parameters for morin hydrate (MH) after oral administration of the MH-loaded formulation F9 compared with those of administered pure MH dispersion as a control.

Pharmacokinetic Parameter	PCM Formulation F9	Morin Hydrate Dispersion
C_max_ (µg/mL)	19 ± 2.6 **	4.8 ± 1.4
T_max_ (h)	6.0 **	2.0
AUC_0–24_ (µg·h/mL)	250 ± 34 **	56 ± 16
AUC_0–∞_ (µg·h/mL)	340 ± 45	77 ± 22

Note: Data are expressed as mean ± SD for *n* = 6; ** significant difference at (*p* < 0.05). Abbreviations: AUC = area under the curve; PCMs = PEGylated chylomicrons.

## Data Availability

All of the data reported herein is included in the Tables and Figures provided and also in the Supporting Information.

## Supplementary Materials

The following supporting information can be downloaded at: https://www.mdpi.com/article/10.3390/pharmaceutics14050905/s1, Figure S1: Particle size distribution determination of the MH dispersion; Figure S2: In-vitro drug release study of F9 in pH 1.2 acidic media. The pH utilized in release study was 7.4 to simulate that of the physiological fluid. At pH 1.2 there is initially greater stability; Figure S3: Experimental data for the concentration profiles of Morin hydrate versus time after oral administration of optimized PCM formula *Left*: F9 (*top*) and MH dispersion (*bottom*) to each of six animals with each reagent. The thick red lines are the profiles of the average concentration values at each data point. *Right*: MERS-CoV plaque assays ± S.D. Table S1: The effect of storage on the physical characteristics of optimized F9 preparation. Table S2: Computer-aided ADMET parameters of morin hydrate.
