# Supplementary material for: Inclusion of a Phytomedicinal Flavonoid in Biocompatible Surface-Modified Chylomicron Mimic Nanovesicles with Improved Oral Bioavailability and Virucidal Activity: Molecular Modeling and Pharmacodynamic Studies
Source: Pharmaceutics. 2022 Apr 21;14(5):905. doi: 10.3390/pharmaceutics14050905 (PMC9144278; doi:10.3390/pharmaceutics14050905)
Supplement: Supplementary file 1 [file pharmaceutics-14-00905-s001.zip › pharmaceutics-1647755-supplementary.pdf]

Supporting Information.

Inclusion of a Phytomedicinal Flavonoid in Biocompatible Surface-Modified Chylomicron Mimic Nanovesicles with Improved Oral Bioavailability and Virucidal Activity: Molecular Modeling and Pharmacodynamic Studies

Mohamed Y. Zakaria [1], Paris E. Georghiou [2,*], Joseph H. Banoub [2,3] and Botros Y. Beshay [4]

Table S1: The effect of storage on the physical characteristics of optimized F9 preparation.

Parameter	Fresh F9	F9 post-3M at 4°C	F9 post-3M at 25°C
**EE%**	89.6±2.9	87.8±1.2	86.1±1.8
**PS**	227.4±13.5	229.1±10.2	231.5±5.4
**Q8h**	78.9±6.7	80.1±8.5	82. 3 ±5.7

Note: All values exploited as mean±SD (n=3), @ non-significant difference at (P>0.05) compared to that of fresh F9]. *Abbreviations:* EE%, entrapment efficiency percentage; PS, particle size; Q8h, % of MH released after 8 h.

Table S2: Computer-aided ADMET parameters of morin hydrate.

**BBB_Lev**[a]	4	**CYP2D6 Prob**[e]	0.4
**Absorption Lev**[b]	2	**CYP2D6** [f]	0
**AQ SOL Lev**[c]	2	**Alog P98**[g]	1.63
**Hepatotox**[d]	1	**ADMET PSA Lev**[h]	140.30

Notes.

a: Blood brain barrier level: 4= undefined, 2= medium penetration, 1= high penetration.

b: Absorption level: 3= very low absorption, 2= low absorption, 1= moderate, 0= good absorption.

c: Aqueous solubility level: 4= optimal, 3= good, 2= low solubility, 1= very low but soluble, 0= extremely low.

d: Hepatotoxicity level: 1= toxic, 0= nontoxic.

e: CYP2D6 inhibition probability.

f: CYP2D6 inhibition: 1= likely to inhibit, 0= non inhibitor.

g: Alog P98: compounds must have log p value not greater than 5.0 to attain reasonable probability of being well absorbed.

h: PSA Lev (polar surface area level): compounds with PSA > 140 have poor bioavailability

Results

			Size (d.nm):	% Intensity:	St Dev (d.n...
Z-Average (d.nm):	672.3	Peak 1:	1176	60.1	310.7
PdI:	0.576	Peak 2:	209.7	32.9	55.20
Intercept:	0.896	Peak 3:	57.16	7.0	10.70
Result quality :	Refer to quality report				

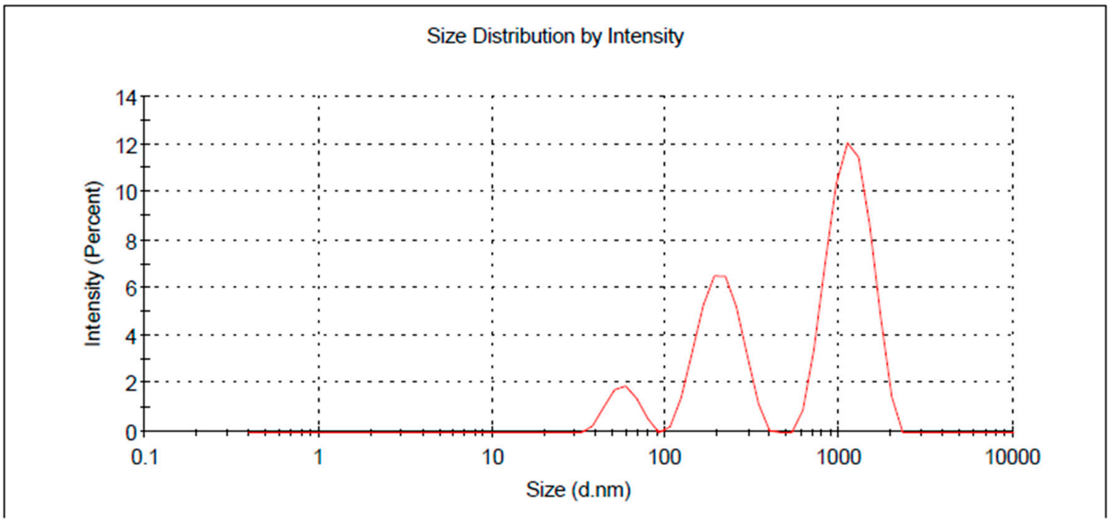

Figure S1. Particle size distribution determination of the MH dispersion.

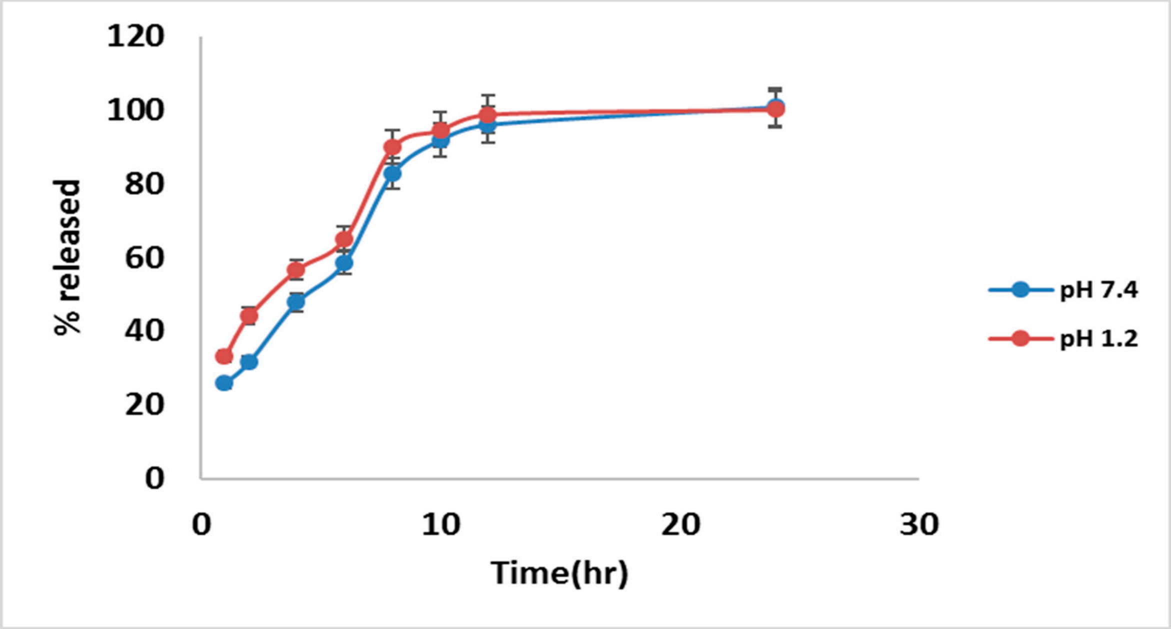

Figure S2. In-vitro drug release study of the optimal MH-Loaded PCM Formulation F9 in pH 1.2 acidic media and at pH 7.4 . The pH utilized in the release study was 7.4 to simulate that of the physiological fluid. At pH 1.2 there is initially greater stability.

For F9:

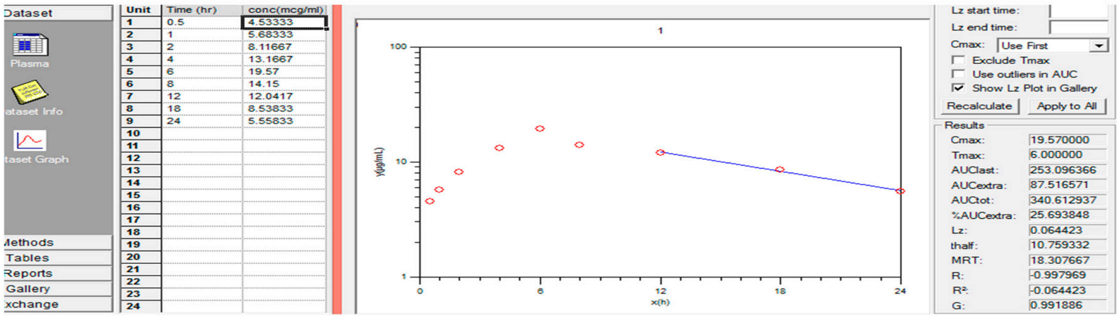

MH-dispersion:

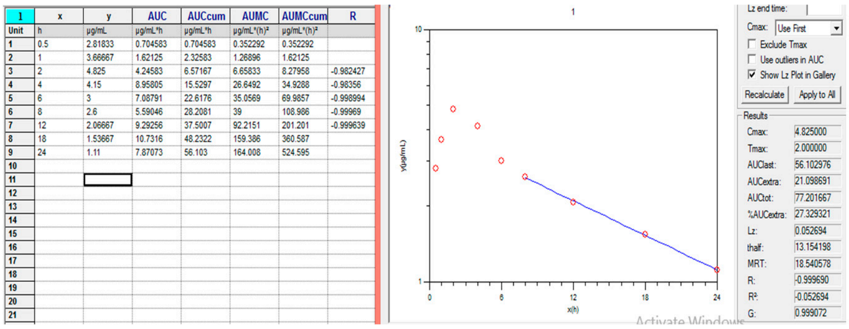

Time	Formula						Average	SD
0.5	4.1	4.6	6.6	2.8	5.8	3.3	**4.5**	1.5
1.0	5.3	4.2	7.4	5.8	6.9	4.5	**5.7**	1.3
2.0	7.7	9.6	11.1	6.2	8.9	5.2	**8.1**	2.2
4.0	12.9	13.6	15.3	11.3	14.1	11.8	**13.2**	1.5
6.0	17.5	20.1	16.4	21.2	18.8	23.4	**19.6**	2.5
8.0	12.2	17.4	11.3	12.9	14.6	16.5	**14.2**	2.4
12.0	10.4	14.3	9.8	10.9	12.7	14.2	**12.0**	2.0
18.0	8.7	9.9	7.5	8.2	9.7	7.2	**8.5**	1.1
24.0	5.4	6.3	4.8	5.9	6.8	4.2	**5.6**	1.0

Time	MH						Average	SD
0.5	1.41	3.50	2.20	4.30	2.60	2.90	**2.8**	1.0
1.0	2.80	3.80	5.10	2.30	4.50	3.50	**3.7**	1.0
2.0	3.20	3.90	5.75	7.20	4.20	4.70	**4.8**	1.4
4.0	3.20	4.20	2.60	5.70	4.40	4.80	**4.2**	1.1
6.0	3.10	2.40	1.90	2.60	3.80	4.20	**3.0**	0.9
8.0	2.80	2.10	1.60	2.40	3.10	3.60	**2.6**	0.7
12.0	1.80	1.60	1.30	2.10	2.70	2.90	**2.1**	0.6
18.0	1.40	1.10	0.87	1.60	2.05	2.20	**1.5**	0.5
24.0	0.85	0.67	0.54	1.30	1.65	1.65	**1.1**	0.5

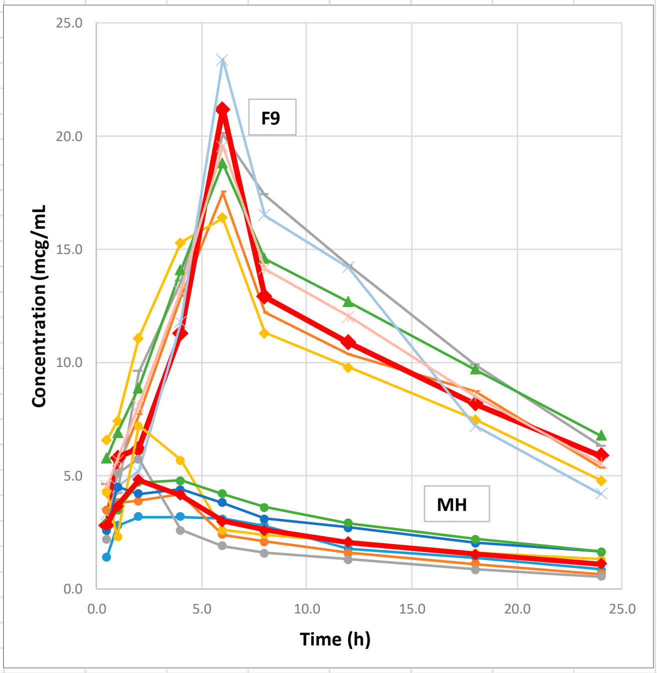

The heavy red lines are the Average Values

Figure S3. Experimental data for the concentration profiles of morin hydrate *vs* time after oral administration of optimized F9 PCM formula *Left:* F9 (*top*) and MH dispersion (*bottom*) to each of six animals with each reagent. The thick red lines are the profiles of the average concentration values at each data point. *Right:* . MERS-CoV plaque assays ± S.D.
